# Tracing the invasion of a leaf-mining moth in the Palearctic through DNA barcoding of historical herbaria

**DOI:** 10.1038/s41598-022-08894-7

**Published:** 2022-03-24

**Authors:** Natalia I. Kirichenko, Evgeny V. Zakharov, Carlos Lopez-Vaamonde

**Affiliations:** 1 Federal Research Center «Krasnoyarsk Science Center SB RAS», Sukachev Institute of Forest, Siberian Branch of Russian Academy of Sciences, Krasnoyarsk, Russia; 2 Siberian Federal University, Krasnoyarsk, Krasnoyarsk Russia; 3 Canadian Center for DNA Barcoding, Centre for Biodiversity Genomics, University of Guelph, Guelph, Canada; 4 Department of Integrative Biology, College of Biological Science, University of Guelph, Guelph, Canada; 5 URZF, INRAE, Orleans, France; 6 IRBI, UMR 7261, CNRS-University of Tours, Tours, France

**Keywords:** Entomology, Invasive species, Next-generation sequencing, Ecology, Genetics, Zoology

## Abstract

The lime leaf-miner, *Phyllonorycter issikii* is an invasive micromoth with an unusually higher number of haplotypes in the invaded area (Europe, Western Siberia) compared to its putative native region (East Asia). The origin of the genetic diversity in the neocolonized region remains unclear. We surveyed over 15 thousand herbarium specimens of lime trees (*Tilia* spp.) collected across the Palearctic over a period of 252 years (1764–2016) looking for preserved larvae within the archival leaf mines. We found 203 herbarium specimens with leaf mines of *Ph. issikii* collected in East Asia, one of them dating back to 1830, i.e. 133 years before the description of the species. In contrast, only 22 herbarium specimens collected in the West Palearctic in the last three decades (1987–2015) carried leaf mines. DNA barcoding of archival specimens revealed 32 haplotypes out of which 23 were novel (not known from modern populations) and found exclusively in East Asia. Six haplotypes are shared between both native and invaded areas and only two were responsible for the recent invasion of the Western Palearctic. The remarkable number of newly discovered haplotypes in archival populations supports East Asia as the native region and the source area of invasion.

## Introduction

Herbarium specimens collected over the centuries in different biogeographic regions have a great value for science^1,2^. They represent not only an important source of information for botanists^3^, but also provide entomologists with paramount evidences on past diversity, distribution, abundance, and trophic associations of insects^4–9^. Despite the fact that botanists tend to avoid collecting damaged plant parts to herbaria^10^, the traces of endophagous insects, in particular leaf mines (i.e. cavities of different shapes made by larvae of some insects that feed inside leaf lamina) are often present in pressed plant specimens^11^. Some of those leaf mines in pressed leaves contain larvae and pupae of leaf-mining insects that have been used in studies on population dynamics^4^, species extinctions^12,13^ and invasion biology^5^.

Here we focus on an invasive leafminining micromoth, *Phyllonorycter issikii* (Kumata, 1963) (Lepidoptera: Gracillariidae), the only species of this genus whose larvae mine exclusively lime *Tilia* spp. (Malvaceae) leaves in the Palearctic. In the last few decades, this species, which was originally known from East Asia (Japan, Korea, Russian Far East)^14–17^, invaded the Western Palearctic and became a pest of lime trees^18–22^. In a phylogeographic analysis of COI DNA barcodes of 334 *Ph. issikii* specimens freshly collected in nature and urban ecosystems across the Palearctic (246 individuals sampled in the invaded region and 88 in the native region), we discovered a total of 31 haplotypes^20^, out of which 23 haplotypes were present in the invaded area (Europe, West Russia and Siberia), whereas only 10 haplotypes were detected in the putative native range in East Asia (the Russian Far East, Japan, South Korea, and China). Only two out of 31 haplotypes (i.e. H1 and H8) were shared between native and invaded areas^20^. Finding three times more haplotypes in the invaded region than in the native one is unusual since often populations of recently introduced non-native insects show the opposite result due to population bottlenecks associated with the process of invasion^5^. The presence of unique haplotypes in the invaded area of *Ph. issikii* not yet detected in the native region could simply be an artifact of insufficient sampling in East Asia. Indeed only 88 specimens from East Asia were sequenced compared with 246 specimens from the invaded region^20^.

Increasing sampling effort in the native region would have implied organizing field expeditions across East Asia to collect fresh material of *Ph. issikii* requiring considerable funding which was not available. Alternatively, one can study international historical herbaria looking for immature stages of *Ph. issikii* found in old mines on pressed leaves in *Tilia* specimens potentially adding a historical dimension to the know invasion pattern^20^. In addition, recent advances in DNA sequencing allow DNA barcoding of old museum specimens^23^ and reveal new haplotypes not yet discovered in collections of fresh material from nature. Indeed, DNA barcoding has already been used to analyse haplotype diversity of leaf-mining larvae found in pressed leaves of horse chestnut trees confirming the Balkans as the native area of the invasive horse-chestnut leaf miner, *Cameraria ohridella* Deschka and Dimić, 1986 (Lepidoptera: Gracillariidae)^5^.

Here, we surveyed numerous pressed lime specimens stored in European herbaria and collected across the Palearctic over the last 253 years. We also took advantage of the recent progress in DNA barcoding of samples with degraded DNA^23^ to characterize COI barcodes of larvae and pupae preserved inside archival leaf mine samples. By increasing the number of sequenced specimens, we would expect to find most of new haplotypes in archival samples collected in East Asia region reducing the difference in haplotype diversity between invaded and native regions. We also expect to find archival leaf mines in herbaria specimens in Eastern Asia well before the beginning of the invasion. In contrast, in the neocolonized region we would expect to find archival leaf mines only in samples collected after the start of the invasion and do not expect to discover many more new haplotypes than the 23 already known from the invaded area^20^. Finally, we also examined herbaria samples of *Tilia* collected in North America between 1850 and 2010 to see whether *Ph. issikii* occurred in the Nearctic but had been overlooked.

## Results

### Detection of archival *Phyllonorycter* mines in historical herbaria

**Figure 1:**
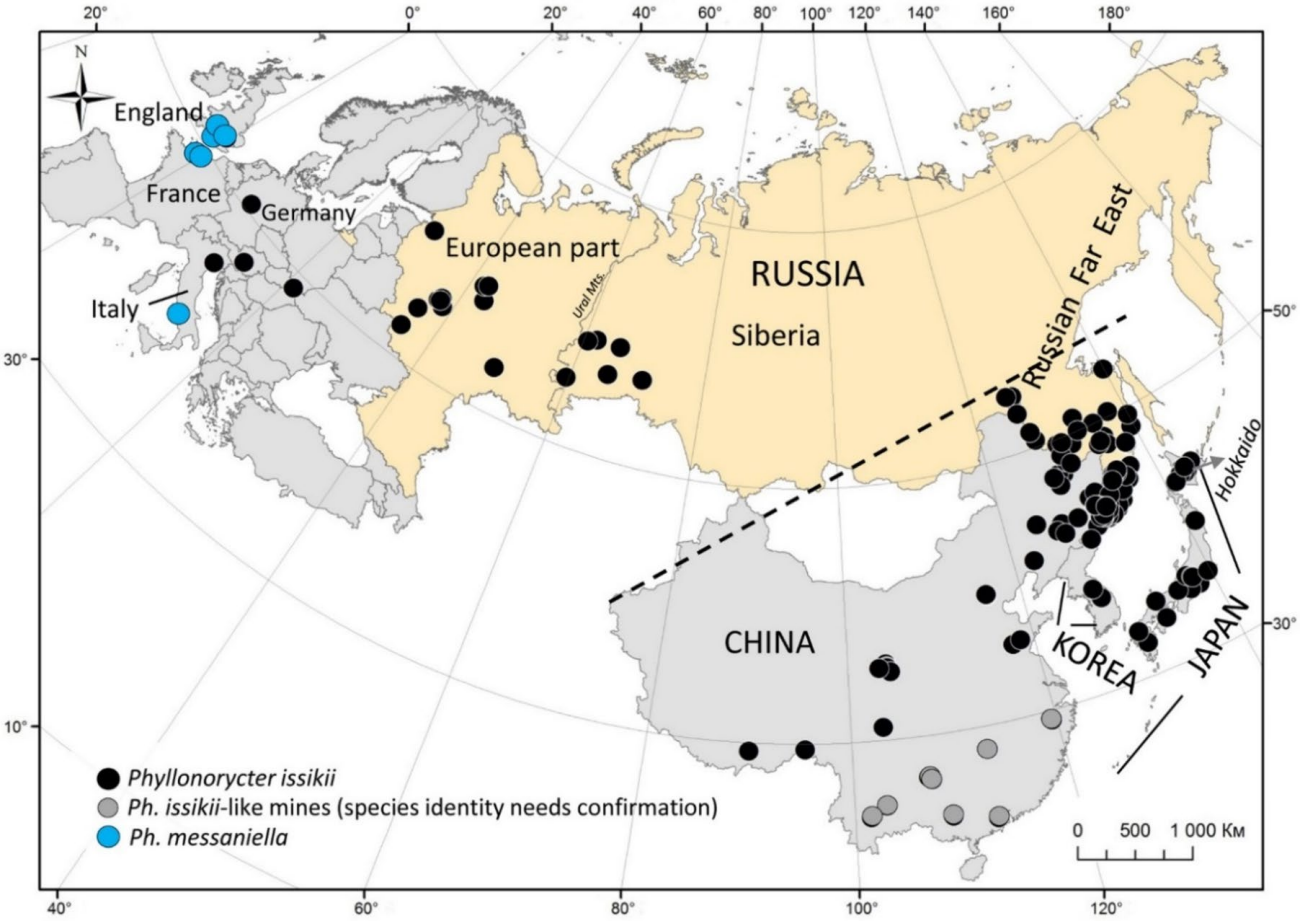
The localities where herbarium specimens of *Tilia* spp. carrying *Phyllonorycter* mines were collected in the Palearctic in the last 253 years. The dotted line divides *Ph. issikii* range to native (below the line) and invaded (above the line). The map was generated using ArcGIS 9.3 (Release 9.3. New York St., Redlands, CA. Environmental Systems Research Institute, http://www.esri.com/software/arcgis/eval-help/arcgis-93).

Only 1.5% (225 out of 15,009) of herbarium specimens of *Tilia* spp. examined from the Palearctic contained *Ph. issikii* leaf mines. These 225 herbarium specimens occurred in 185 geographical locations across the Palearctic, with the westernmost point in Germany (Hessen; the herbarium specimen dated by 2004) to the most eastern locations in Japan (on the island of Hokkaido; 1885–1974) (Fig. 1).

**Figure 2:**
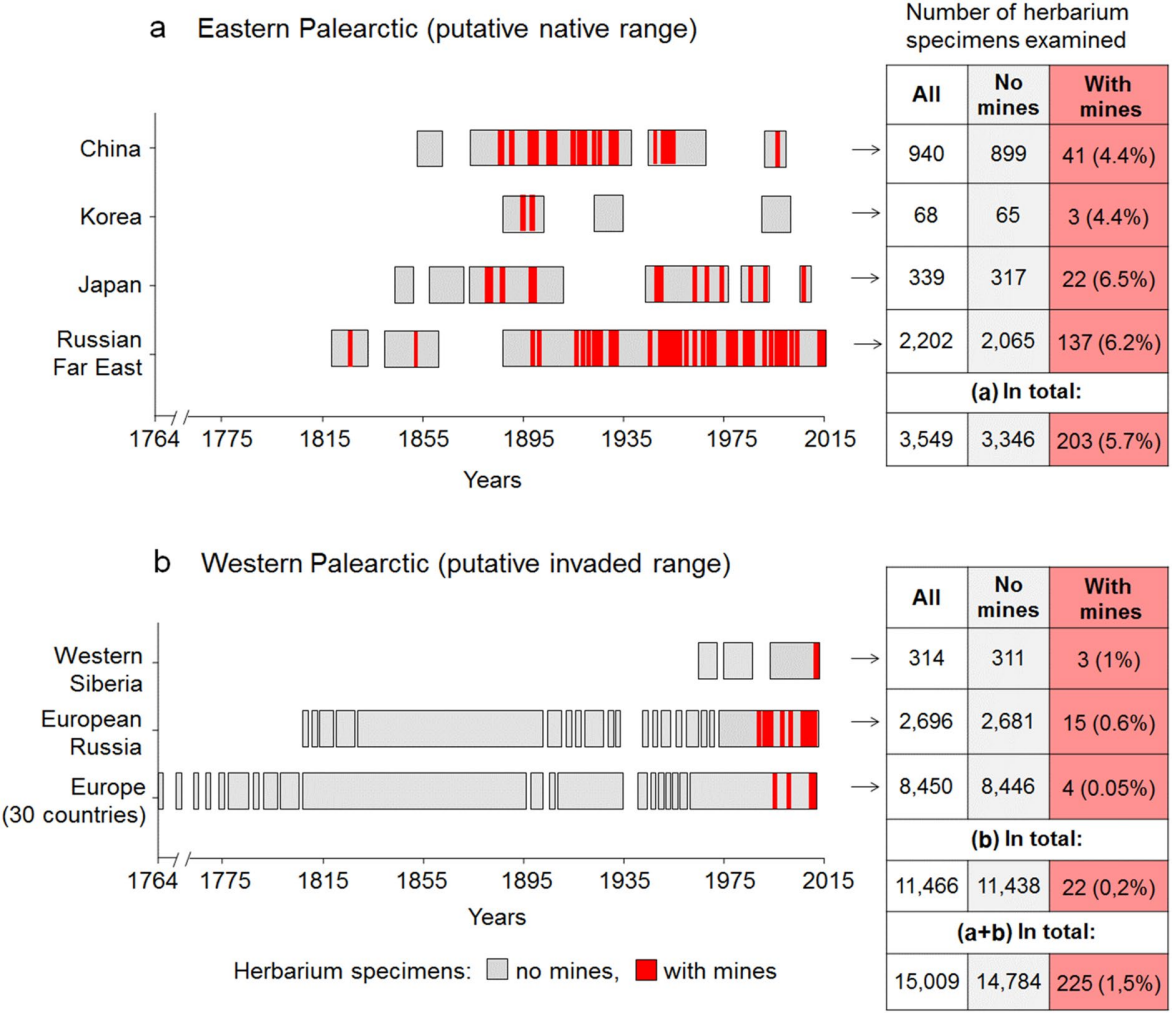
The presence of *Phyllonorycter issikii* mines in the herbarium specimens collected in the putative native (**a**) and invaded (**b**) ranges over the past 253 years (1764–2016). The number of herbarium specimens with and without mines and the percentage of the specimens with mines in each region or country from all herbarium specimens examined in a region or country (in brackets) are given next to each graph. The total number of herbarium specimens, including those with and without mines, is given for Eastern (a) and Western Palearctic (b) separately and altogether (a + b).

Most specimens with leaf mines (90%; 203/225) originated from Eastern Palearctic, in particular from the Russian Far East (RFE) (67.5%, 137/203) (Fig. 2a). In some cases, leaves were severely attacked, carrying up to 12 mines per leaf (as documented in the Russian Far East in 1930s–1960s). On the other hand, we found only 22 herbarium specimens with mines (10%; 22/225) from the putative invaded region in Western Palearctic, with the majority of herbarium specimens with mines (7% 15/225) from European Russia (Fig. 2b).

The average number of leaf mines per herbarium specimen found in native (5.68 ± 0.77) and invaded regions (6.09 ± 1.70) was not significantly different (Mann–Whitney U-test: U = 20,145; Z = 0.43; *p* = 0.43). However, the infestation rate by *Ph. issikii,* i.e. percentage of leaves with mines per herbarium specimen was statistically higher in the West than in the East: 35% ± 8.19 versus 23% ± 1.94 (Mann–Whitney U-test: U = 1339; Z = 2.30; *p* = 0.02).

Leaf mines from the East were significantly older than those from the West (Mann–Whitney U-test: U = 81; Z =  − 4.4; *p* < 0.001). Indeed, *Ph. issikii* mines from the West were found on herbarium specimens collected exclusively in the last three decades (1987–2015) (Figs. 2a, S1), whereas in East Asia, they were detected in herbarium samples dating back to 1830 (Figs. 2b, S1). The oldest mines were revealed in the pressed lime leaves sampled in the RFE: in Primorsky Krai, Sikhote-Alin Nature Reserve in 1830 (one empty mine), followed by the finding in Amur Oblast (village Busse, 1.5 km from the border with the Chinese province Heilongjiang) in 1859 (the mine contained a larva that was identified as *Ph. issikii* by DNA barcoding). These archival mines were 191- and 162-year-old respectively, and thus they dated 133 and 104 years before *Ph. issikii* description from Japan (1963). The time lag between the earliest mine record on the East (1830) and that on the West (1987) was 157 years.

We found six European herbarium specimens with eleven mines of the polyphagous moth *Phyllonorycter messaniella* (Fig. 1; Table S1). Seven out of eleven mines were assigned to *Ph. messaniella* by their morphology, i.e. presence of one distinct longitude fold on the epidermis covering the mines. However, the remaining four mines were at an early stage of development and the fold was not visible therefore DNA barcoding was used to identify them (see “Results” section below).

None of the 683 North American herbarium specimens of *Tilia* contained *Ph. issikii* mines. However, we found 37 specimens (5.4%) with 88 leaf mines of *Ph. lucetiella* and *Ph. tiliacella* with the specimens’ ages ranging from 11 to 197 years old (Table S2).

### Distribution of *Ph. issikii* in the past as inferred from herbaria data

#### Eastern Palearctic (putative native range)

Out of the 3549 herbarium samples examined from the Eastern Palearctic 203 (5.7%) carried characteristic leaf mines. We found *Ph. issikii* mines on 41 out of 940 herbarium specimens (4.4%) in China, 3 out of 68 (4.4%) in Korea, 137 out of 2202 (i.e. 6.2%) in the RFE, and 22 out of 339 (6.5%) in Japan (Fig. 2a).

**Figure 3:**
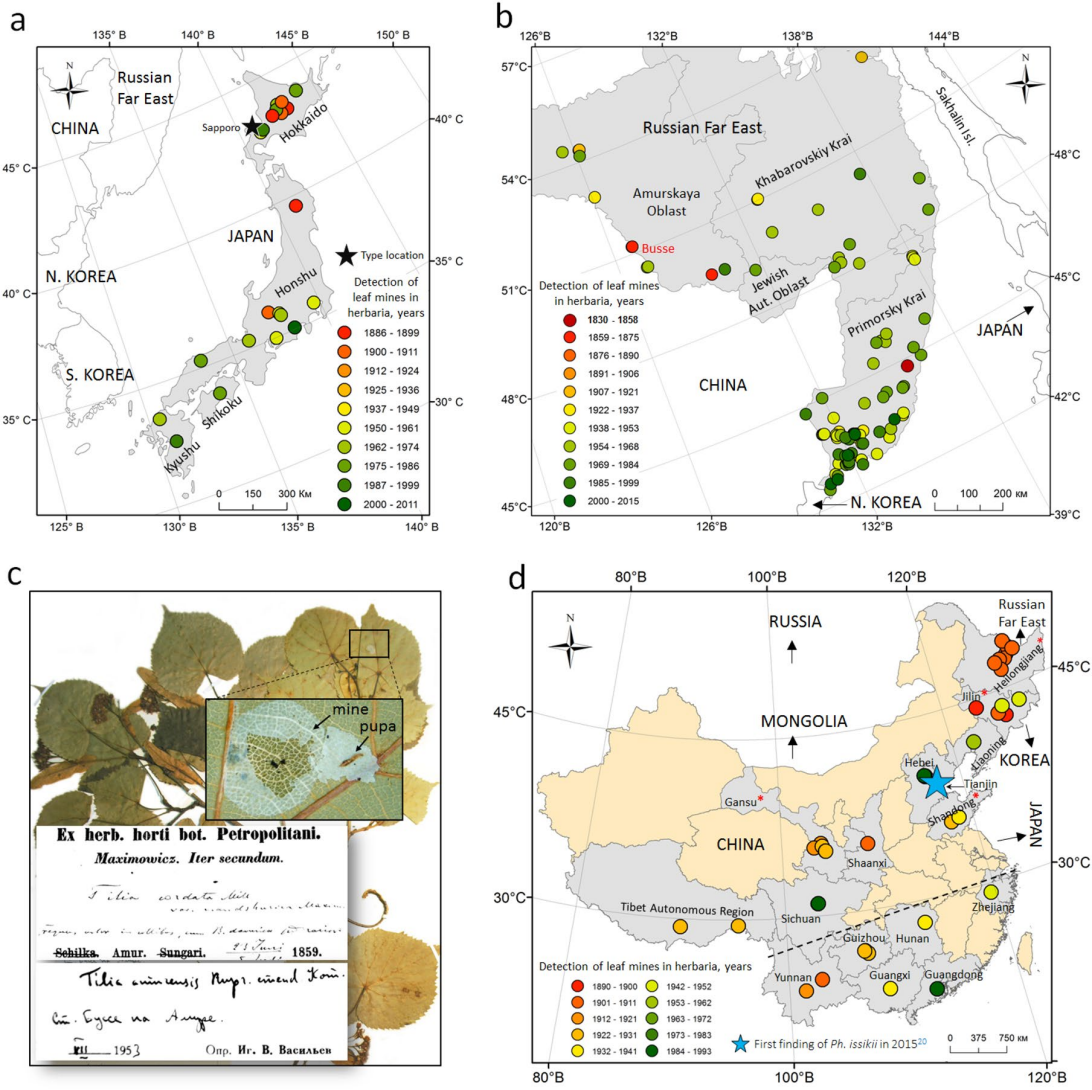
Past distribution of *Phyllonorycter issikii* in Japan (**a**), the Russian Far East (**b**), and China (**d**) based on findings of leaf mines in herbaria collected in 1859–2015. The village Busse, 1.5 km from the border with the Chinese province Heilongjiang, is the location of the earliest finding of *Ph. issikii* mines (1859) confirmed by DNA barcoding (**b**, **c**). In China (**d**), the provinces where typical mines were found in herbaria are shaded in gray; in the provinces marked by an asterisk, the identification of *Ph. issikii* archival specimens was confirmed by DNA barcoding. The dotted line shows a schematical border between the Palearctic (above the line) and the Indomalaya (below the line). The maps were generated using ArcGIS 9.3 (Release 9.3. New York St., Redlands, CA. Environmental Systems Research Institute, http://www.esri.com/software/arcgis/eval-help/arcgis-93). Photographs in Fig. (**c**) taken by N. Kirichenko.

In Japan, from where *Ph. issikii* was formally described^14^, the mines were found in herbaria samples collected between 1886 and 2011, from the North to the South (32°–45° N) and from the West to the East (130°–145° E) (Fig. 3a). The earliest finding of *Ph. issikii* mines dated back to 1886 from Hokkaido.

In the RFE, herbarium samples with leaf mines were collected between 1830 and 2005 in 137 localities in Primorsky Krai, Khabarovsk Krai, Jewish Autonomous Oblast and Amur Oblast (Fig. 3b). The characteristic mines were also found in pressed lime specimens from the islands Russky, Popov and Askold (sampling years 1973–1998), from where *Ph. issikii* was not earlier documented in the literature. According to the findings of mines in herbaria, the historical range of *Ph. issikii* in the RFE covered a significant area reaching the latitudes of 42°–54.5° N and the longitudes of 126°–139° E. Importantly, in the RFE the mines of *Ph. issikii* were regularly found in the herbaria from the localities next to the border with China and North Korea (Fig. 3b,c).

In China, the typical mines on pressed lime specimens were for the first time found in 15 provinces: from Heilongjiang in the northeast to Yunnan, Guangdong and Guangxi Zhuang Autonomous Region in the south, i.e. on the territory between 47°–32° N and 105°–125° E (Fig. 3d), whereas the species was known from China only by a single record from Tianjin^20^. The age of the herbarium specimens on which those mines were detected in China varied from 28 to 131 years (the herbarium specimens were collected in 1890–1993) (Fig. 3d). The oldest specimens with leaf mines originated from the northeastern provinces Jilin (1896) and Heilongjiang (1902).

In Korea, only six mines were found in three lime specimens: one mine in two herbarium specimens collected in 1900–1902 during the Korean-Sakhalin expedition by the Imperial Russian Geographical Society and the other four mines on the specimen dated by 1909 without indication of the exact sampling location on the label.

#### Western Palearctic (putative neocolonized range)

In Western Palearctic, only 0.2% of herbarium samples examined (22/11,466) had *Ph. issikii* leaf mines. In Western Siberia, 1% of herbarium samples (3/314) had *Ph. issikii* mines, followed by European Russia and Europe (30 countries sampled), where only 0.6% (15/2696) and 0.05% (4/8450) of the specimens, respectively, had the mines (Table 1).

**Table 1:** Host plants and level of attack (% of mined leaves) of *Phyllonorycter issikii* found in 22 herbarium samples collected across the Western Palearctic between 1987 and 2016.

№	Country, region*	Location** and biotope	Host plant***	Number of			Year
				Mines	Leaves with mines [in %***]	Leaves in herbarium specimen	
**EUROPE**							
1	Austria, Styrian	—; [f]	*T.c.*	10	6 [11]	54	2015
2	Germany, Hessen	—; [f]	*T.c.*	2	2 [9]	22	2014
3	Italy, Veneto	—; [f]	*T.p.*	7	5 [45]	11	2011
4	Slovakia	vil. Trebejov; [f]	*T.c.*	2	2 [11]	18	2006
**RUSSIA: European part**							
5	Chelyabinsk Obl.	Zlatoust; [f]	*T.c.*	3	3 [30]	10	1987
6	Kaluga Obl.	vil. Klykovo; [p]	*T.p.*	1	1 [11]	9	2008
7	Kostroma Obl.	vil. Kozlovo; [f]	*T.c.*	12	7 [28]	25	2014
8	Kostroma Obl.	vil. Bogorodskoe; [f]	*T.c.*	28	18 [100]	18	2014
9	Kostroma Obl.	vil. Seraphikha; [f]	*T.c.*	5	5 [26]	19	2013
10	Kursk Obl.	Zheleznogorsk; [p]	*T.c.*	26	6 [100]	6	2011
11	Leningrad Obl.	Pavlovsk; [p]	*T.p.*	1	1 [11]	9	2000
12	Moscow Obl.	Moscow, Lublino; [p]	*T.c.*	2	2 [14]	14	2012
13	Moscow Obl.	vil. Gorodishe	*T.p.*	3	2 [100]	2	2015
14	Moscow Obl.	Moscow [p]	*T.p.*	2	2 [18]	11	2012
15	Moscow Obl.	Moscow [p]	*T.p.*	17	8 [100]	8	2006
16	Moscow Obl.	Moscow [p]	*T.p.*	1	1 [14]	7	2006
17	Samara Obl.	Zhiguli Nat. Reserve; [f]	*T.c.*	7	6 [33]	18	1990
18	Sverdlovsk Obl.	Sverdlovsk suburb [f]	*T.c.*	1	1 [8]	13	1991
19	Sverdlovsk Obl.	Sverdlovsk suburb [f]	*T.c.*	1	1 [4]	26	1991
**RUSSIA: Western Siberia**							
20	Khanty-Mansi AO	vil. Kumisnkiy, [f]	*T.c.*	1	1 [4]	23	2015
21	Tyumen Obl.	Vikulovskiy district, [f]	*T.c.*	1	1 [7]	15	2007
22	Tyumen Obl.	Isetskiy district, [f]	*T.c.*	1	1 [5]	19	2012

*Obl., Oblast; **—, no data; vil., village; f, forest; p, park; ***T.c., *Tilia cordata*; *T.p.*, *Tilia platyphillos*; ****Percentage of mined leaves was calculated as number of mined leaves over total of examined leaves*.*

None of the other 11,438 herbarium specimens collected before 1987 showed any leaf mines. In European Russia leaf mines were found in herbarium specimens collected between 1987 and 2015 in nine regions: from Leningrad Oblast on the north west to Chelyabinsk Oblast on the east (on the border between Asia and Europe) (Table 1). In Central Europe, in particular Austria, Germany, Italy, and Slovakia, archival leaf mines were found in herbarium specimens sampled between 2006 and 2015 (Table 1). In Western Siberia, the mines were found in pressed limes sampled in Khanty-Mansi Autonomous Okrug and Tyumen Oblast in 2007–2015 (Table 1).

### Host plant use and level of attack of *Ph. issikii* in archival samples

We found archival leaf mines of *Ph. issikii* feeding on 18 different *Tilia* species in the putative native region of East Asia and only on two species of Tilia (*T. cordata* and *T. platyphyllos*) in the putative invaded area in the Western Palearctics (Fig. S2, Table S3).

In the East, 33% of all herbarium specimens with mines were found on *T. amurensis* (74/225) followed by *T. taquetii* (19%, i.e. 42/225) and *T. mandshurica* (11%, 24/225) (Fig. S2, Table S3). Occasionally, mines were detected on other 15 different East Asian lime species (Fig. S2, Table S3). We documented *Ph. issikii* mines on five novel host plants in China: *T. chinensis, T. intonsa, T. leptocarya, T. miqueliana*, and *T. paucicostata* (Fig. S2). Among these five species, *T. leptocarya* is presently considered as a synonym of *T. endochrysea.* In the West, *Ph. issikii* mines were found in the herbarium specimens sampled on *T. cordata* (15/225, i.e. 7%) and *T. platyphyllos*. In European Russia and Western Siberia, they were revealed only on *T. cordata* (7/225, i.e. 3%) (Fig. S2).

### DNA barcoding of archival samples

DNA barcodes were obtained for 88 out of 93 (i.e. 95%) archival larvae and pupae of *Phyllonorycter* species dissected from 7 up to 162-year-old lime herbarium (Table S4). The remaining five archival larvae and pupae (four 74–125-year-old specimens from the Palearctic, and one 171-year-old specimen from the Nearctic) failed to produce sequences. Among 88 sequenced specimens 73 specimens originated from the Palearctic (dated from 1859–2014) and 15 specimens were from the Nearctic (dated from 1894–2010) (Table S4).

**Figure 4:**
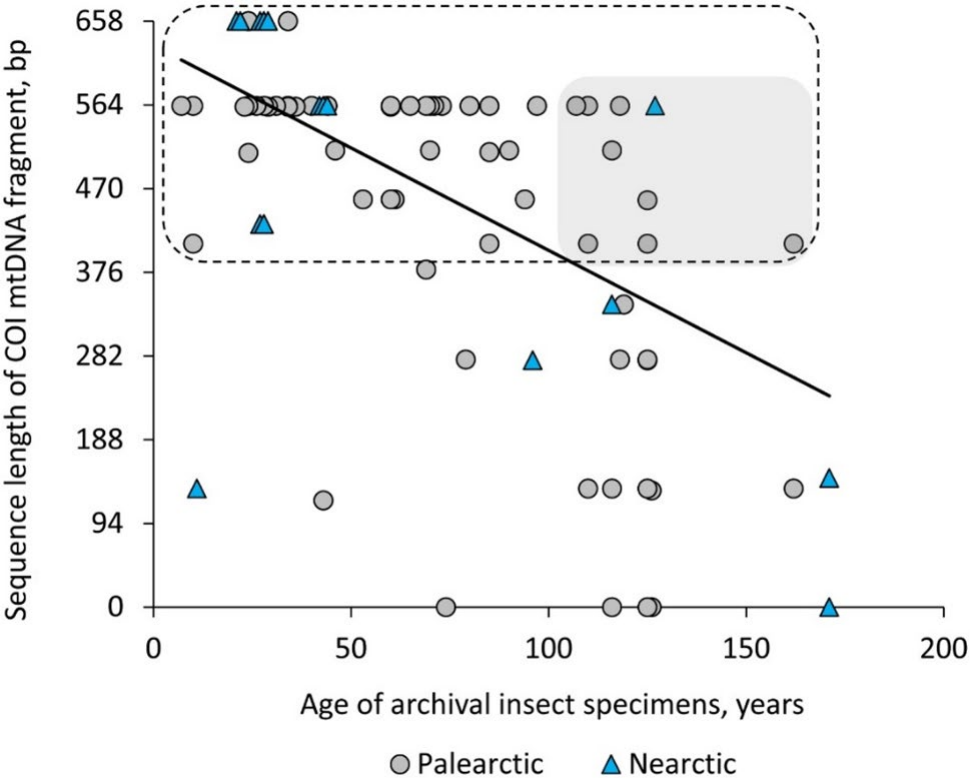
The relationship between the length of sequenced COI mtDNA fragment and the age of the archival *Phyllonorycter* specimens dissected from the mines in herbaria sampled in the Palearctic and Nearctic between 1850 and 2016. A linear regression is shown in the figure; y =  − 2.4x + 630, R^2^ = 0.32, N = 93, *p* < 0.05. The dashed frame indicates the 71 archival specimens (from 7 to 162 years old) with relatively long sequences (> 60% of the total length); eight samples of over one century old that were successfully sequenced are highlighted within the grey rectangle.

The length of recovered COI sequences ranged from 120 base pairs to 658 bp (Fig. 4, Table S5).

The sequence length for the archival insect specimens was negatively and significantly correlated with the specimen age (R^2^ = 0.32, N = 93, *p* < 0.05). Relatively long sequences (> 60% of the total length, i.e. the sequenced length > 400 bp) were obtained for 71 archival specimens that were between 7 and 162 years old (Fig. 4, the points in dashed frame) (Table S4). Nine of these 71 specimens were over one century old (106–162-year-old): eight originated from the Palearctic and one from the Nearctic (Fig. 4, the points in gray cloud).

**Figure 5:**
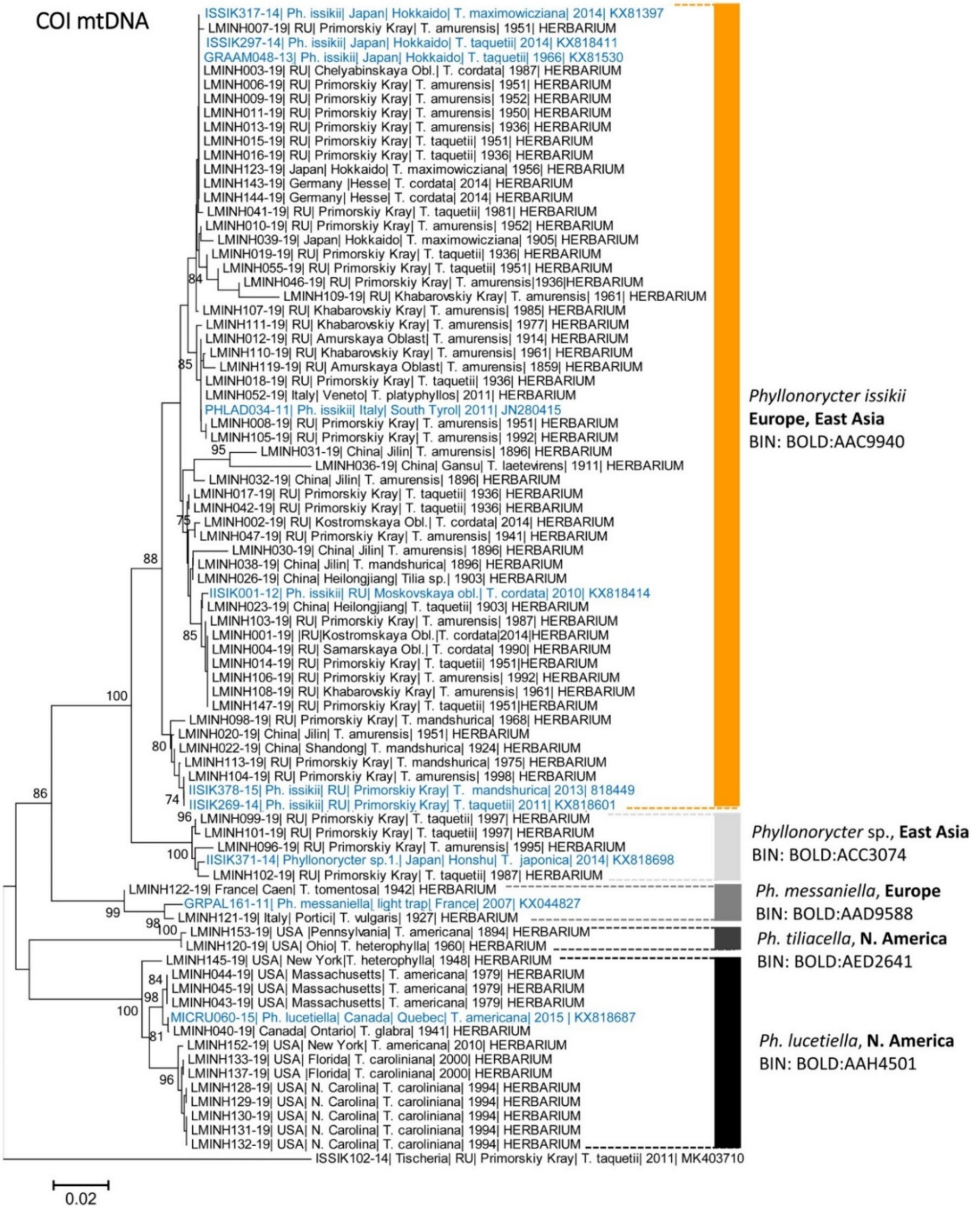
A maximum likelihood tree of 81 COI sequences of *Phyllonorycter* spp. Overall, 71 archival sequenced specimens were dissected from herbaria collected in the Palearctic and the Nearctic in 1859–2014 and ten specimens (highlighted in blue) originated from the modern range^20^. The tree was generated with the K2P nucleotide substitution model and bootstrap method (2500 iterations), *p* < 0.05. Each specimen is identified by its unique Process ID | country | region | host plant | sampling year | GenBank accession number (for modern sequences) or an indication of the source of the material (HERBARIUM) for archival specimens. Each genetic cluster is specified by its Barcode Index Number (BIN) (given next to each cluster). Branch lengths are proportional to the number of substitutions per site.

In the Palearctic, the oldest successfully DNA barcoded *Ph. issikii* specimen (obtained sequence length 408 bp) was a 162-year-old larva dissected from the leaf mine on *Tilia amurensis* from the RFE (village Busse, Amur Oblast, the year 1859), sequence ID LMINH119-19 (Fig. 5, Table S5). In the Nearctic, the oldest sequenced specimen (obtained sequence length 658 bp) was 127-year-old larva of *Ph. tiliacella* on *T. americana* from USA, Pennsylvania (Fig. 5, Table S5)*.*

The 71 archival sequences represented five distinct genetic clusters, each corresponding to a unique BIN (Fig. 5).

Among them, two BINs with a Palearctic distribution were identified as *Ph. issikii* (number of specimens, N = 50, from 1859–1981) and *Ph. messaniella* (N = 2, from 1927 and 1942), and two BINs from the Nearctic were determined as *Ph. lucetiella* (N = 13, 1941–2010) and *Ph. tiliacella* (N = 2, 1894 and 1960). The fifth BIN was formed by a putative new *Tilia*-feeding species from the RFE and Japan^20^ (N = 4, 1987–1997) (Fig. 5), showing a minimum pairwise K2P distance of 4.79 with *P. issikii* (Table 2).

**Table 2:** Intra- and interspecific genetic divergences in DNA barcode fragments (COI mtDNA) in the archival specimens of lime-feeding *Phyllonorycter* spp. dissected from herbaria in the Palearctic and Nearctic*.

Species, (realm)	Species				
	*Ph. issikii*	*Ph. messaniella*	*Ph. tiliacella*	*Ph. lucetiella*	*Phyllonorycter* sp.
*Ph. issikii* (Palearctic)	[3.1]				
*Ph. messaniella* (Palearctic)	5.75	[0.72]			
*Ph. tiliacella* (Nearctic)	9.69	10.48	[0.18]		
*Ph. lucetiella* (Nearctic)	11.04	7.58	10.48	[1.86]	
*Phyllonorycter* sp. (Palearctic)	4.79	6.11	13.14	11.55	[1.06]

*Kimura two-parameter (K2P) distances; each species pair is provided with minimal pairwise distances; values in square brackets show maximal intraspecific distances. Number of analyzed archival specimens (N): *Ph. issikii* (50), *Ph. lucetiella* (13), *Ph. messaniella* (2), *Ph. tiliacella* (2), *Phyllonorycter* sp. (4).

The BIN of *Ph. issikii* contained 50 archival specimens: 42 from the East and 8 from the West of the Palearctic. In the East, the archival specimens originated from the RFE (32 specimens from 1859–1981), China (8 specimens from 1896–1924) and Japan (2 specimens from 1905–1956) (Fig. 5). On the other hand, in the West, the archival specimens of *Ph. issikii* originated from Europe (2011–2015) and European part of Russia (1987–2015) (Fig. 5).

### Haplotype diversity and phylogeography of *Ph. issikii*

Haplotype diversity in *Ph. issikii* past populations was higher in the East (0.93) than in the West (0.65) (Mann–Whitney U test: U = 6, Z =  − 0.75, *P* < 0.005).

**Figure 6:**
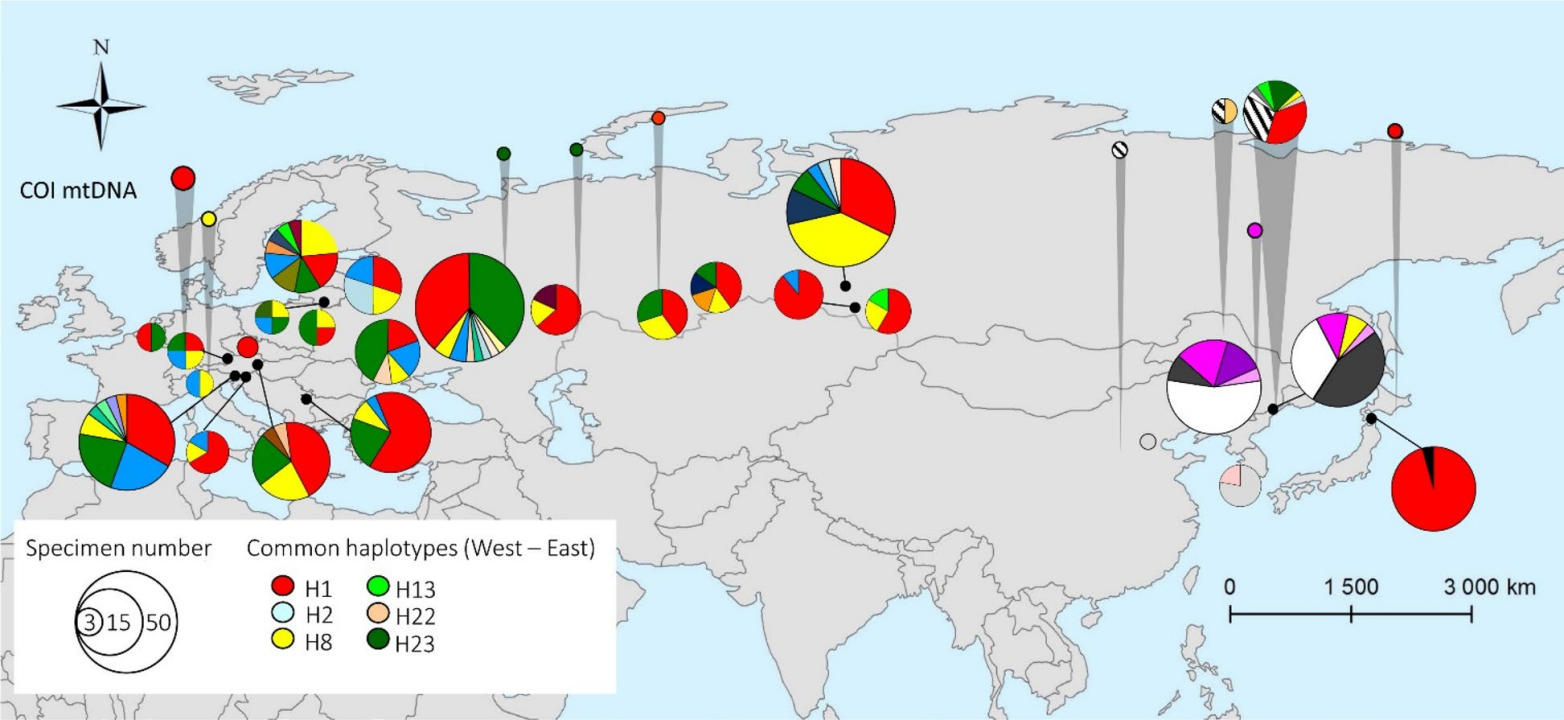
Geographical distribution of historical and modern haplotypes of *Phyllonorycter issikii* found across the Palearctic. The data on the haplotype diversity of *Ph. issikii* in the modern range is in agreement with our previous study^20^. Each pie chart represents a country, except Russia where nearest locations are merged into one pie chat. Each color of pie charts refers to one of the haplotypes found across the Palearctic; the haplotypes sequenced in archival insects are shown in 10 pie charts with grey callouts. Six haplotypes are present both in the East and West Palearctic and are found in both modern and archival samples (see legend); the historical haplotypes that are not present in the modern range are counted altogether into one sector of a circle diagram with line shading. The exact geographical location of historical haplotypes can be found in Tables S5, S6. The map was generated using ArcGIS 9.3 (Release 9.3. New York St., Redlands, CA. Environmental Systems Research Institute, http://www.esri.com/software/arcgis/eval-help/arcgis-93).

**Figure 7:**
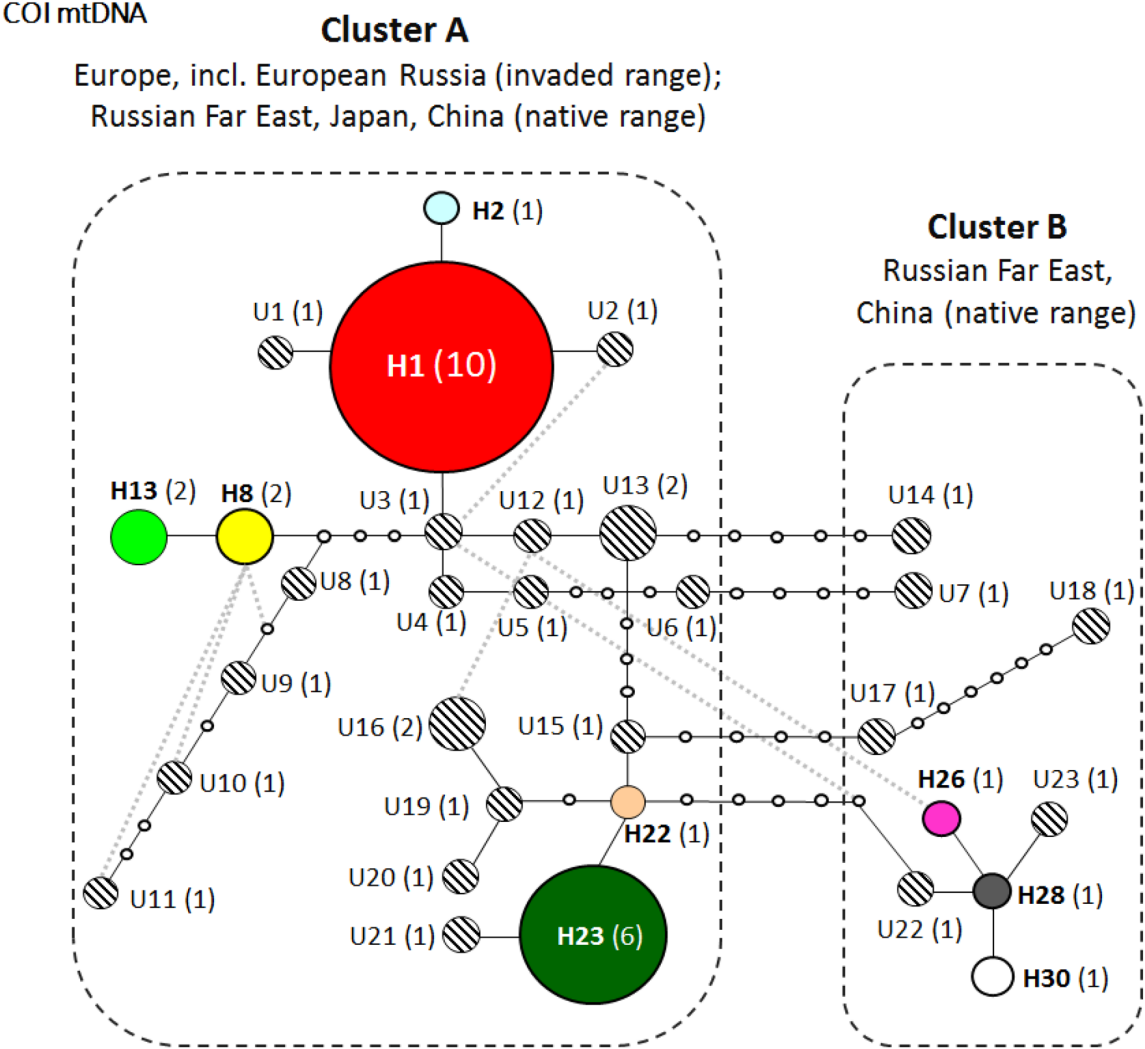
Median haplotype network of *Phyllonorycter issikii* in the Palearctic based on sequencing of 50 archival specimens dissected from historical herbarium collected between 1859 and 2014. Different colors correspond to nine haplotypes (H1, H2, H8, H13, H22, H23, H26, H28, H30) that are also known from modern *Ph. isskii* specimens^20^. Historical unique haplotypes (documented for the first time from archival specimens) are indicated by numbers U1–U23 next to the corresponding circles with shading. Empty tiny circles indicate intermediate missing haplotypes. The number of sequenced individuals is given in brackets next to each haplotype. Each line connecting the circles indicates one mutation step. Dotted rectangles indicate the two main clusters. The geographical distribution of haplotypes is given in Tables S5, S6.

Overall, 32 haplotypes were found among 50 sequenced archival specimens of *Ph. issikii* (Figs. 6, 7, Tables S5, S6). All of them were detected in East Asia, including six which were shared with the West (H1, H2, H8, H13, H22, and H23) and are also present in modern specimens (Fig. 6, Table S5). The two commonest haplotypes among archival sequences were H1 (red) with 30% (15/50) sequences and H23 (green) with 14% (7/50) sequences (Figs. 6, 7). Notably, both haplotypes H1 and H23 are thought to be responsible for the invasion of the Western Palearctic dominating in the modern populations of *Ph. issikii* and widely found both in the Eastern and Western Palearctic^20^. Based on the analysis of archival herbaria, the haplotypes H13 and H23 were for the first time detected in the RFE and H22 in China, and the presence of the most distributed invasive haplotype H1 was confirmed by historical material from Japan and the RFE (Table S5) suggesting the contribution of Russia Far Eastern, Chinese and Japanese populations to the invasion of the moth westwards. Three haplotypes (H26, H28, H30) recorded in the archival *Ph. issikii* specimens from China and the RFE have been already known from East Asia through sequencing the insect specimens sampled in nature in twenty-first century, as per our recent study^20^ (Table S5).

In total, 23 out of 32 haplotypes were found for the first time and exclusively in archival specimens of *Ph. issikii* from East Asia (shown in circles with shading in Fig. 7, Table S6).

The reconstruction of the haplotype network supported the presence of two genetically differentiated clusters (A and B) of *Ph. issikii* in the past range (Fig. 7). Cluster A was formed by haplotypes found in both the western and eastern parts of the Palearctic, whereas Cluster B included haplotypes exclusively found in East Asia (the Russian Far East and China) (Fig. 7). A minimum of four mutations were detected between the nearest haplotypes of clusters A and B, i.e. between U6 (Cluster A) and U7 (Cluster B), and U 15 (A) and U17 (B) (Fig. 7).

## Discussion

Our study confirms that DNA preservation in archival larvae and pupae sealed in tissue of pressed leaves is sufficient for identification of century-old leafmining insect remains. Indeed, we were able to sequence archival larvae and pupae dissected from up to 162-year-old herbaria specimens, with 76% of sequences being 400–658 bp. As expected, sequence length was negatively correlated with the age of insect specimens. A similar negative relationship between sequence length and specimen age was found for museum specimens of geometrid moths (dated up to 157 years old) which were DNA barcoded with Sanger sequencing^24^. The length of recovered sequences also showed a tendency to decrease with the age in DNA barcoded century-old Lepidopteran specimens^25,26^, with higher sequence recovery rate obtained with high-throughput sequencing than Sanger sequencing^25^.

The survey of historical herbaria shows that, as expected, the majority of herbarium specimens with leaf mines were collected in East Asia over a long period of time (starting from 1830) with many collected well before the beginning of the documented population expansion westwards in 1980s^18–20^. In contrast, archival leaf mines coming from Europe and Western Siberia were sampled over a much more recent period (1987–2015) after the start of the invasion. In addition, DNA barcoding analysis of archival larvae discovered in historical herbaria revealed the existence of 32 haplotypes among which 23 haplotypes were unique and only found in the past exclusively in East Asia. Overall, six out of the remaining nine haplotypes, all found in East Asia in the past, are also known from the neocolonized area in Western Palearctic from modern times, and three haplotypes are presently found in the Russian Far East^20^. This new genetic diversity detected in East Asia suggests that the native range of *Ph. issikii* is indeed the Russian Far East, Japan, Korea, and China. Furthermore, the discovery of those 23 unique archival haplotypes brings the total number of haplotypes known for *Ph. issikii* from the previous study (i.e. 31 haplotypes)^20^ to 54, thus, significantly contributing to the knowledge of the species genetic diversity. It makes *Ph. issikii* one of Gracillariidae species with the highest number of known haplotypes and comparable with *Salix*-feeding *Phyllonorycter salictella* for which 56 haplotypes have been documented^20^.

To our big surprise, herbarium material and DNA sequencing analysis revealed broad presence of *Ph. issikii* in China suggesting that the occurrence of the species had been overlooked in the country for decades. Indeed, the species was not known from China before its first record in Tianjin in a previous study^20^ (see also Fig. 3d). Our current survey revealed archival mines in 15 different Chinese provinces, mostly in northeast and central parts of the country. The archival mines from Southern China provinces (refer to Indomalaya) did not differ from those of *Ph. issikii*. However, the species identity could not be proven as no larvae or pupae were found within the mines in herbarium specimens. Thus, further work is needed to confirm the presence of *Ph. issikii* in Southern China.

Some herbarium samples from East Asia collected before the beginning of the invasion were heavily mined suggesting that outbreaks are a natural phenomenon in *Ph. issikii* as in other species of Gracillariidae^5^. Indeed, herbarium specimens (*T. amurensis, T. taqueti*), from the RFE collected between 1934 and 1958 had up to 100% of leaves with mines (up to 13 mines per leaf). Prior to our study, no data was available in the literature recording significant damage by *Ph. issikii* in the native range (East Asia), except observation of an outbreaking density of *Ph. issikii* in Sapporo, Hokkaido (Japan) in 2002^20^. In contrast, outbreaking densities have been regularly recorded in Europe since the first discovery of the species in European Russia in 1985^19,27,28^.

**Figure 8:**
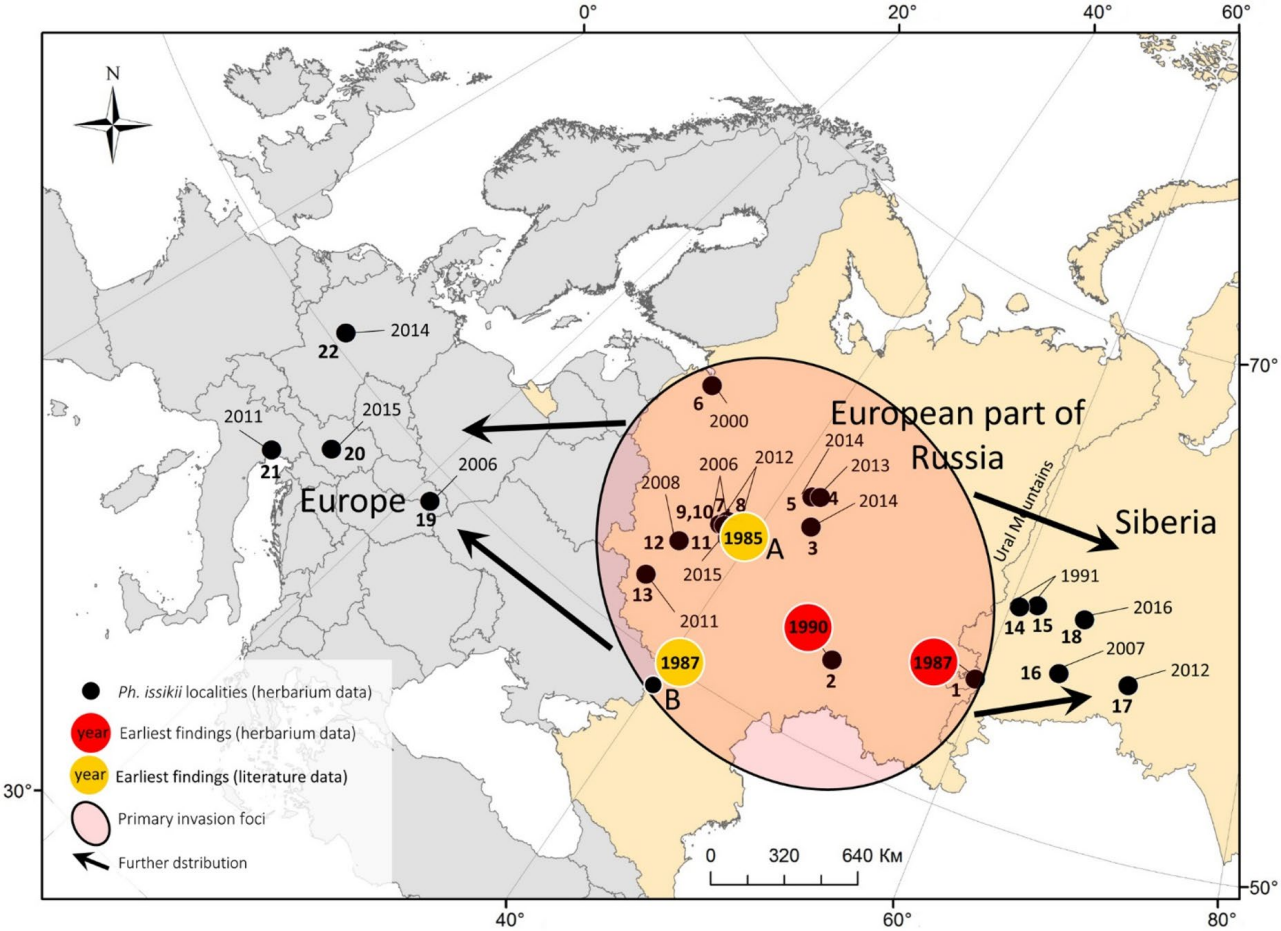
The suggested scenario of *Phyllonorycter issikii* invasion in Western Palearctic based on archival data from the historical herbaria and published records. The dots numbers (1–22) indicate the regions or countries where archival mines were found in herbaria. Next to the dots, the year of collection of herbarium specimens with *Ph. issikii* mines is indicated: 1–18—Russia (1—Chelyabinsk, 2—Samara, 3–5—Kostroma, 6—Leningrad, 7–11—Moscow, 12—Kaluga, 13—Kursk, 14–15—Sverdlovsk, 16–18—Tyumen Oblasts), 19—Slovakia, 20—Austria (Styria), 21—Italy (Veneto), 22—Germany (Hessen). Black arrows show possible directions of *Ph. issikii* expansion from the primary foci. The map was generated using ArcGIS 9.3 (Release 9.3. New York St., Redlands, CA. Environmental Systems Research Institute, http://www.esri.com/software/arcgis/eval-help/arcgis-93).

*Phyllonorycter issikii* is currently known widely in Europe and European Russia, almost everywhere where limes are present^18–20^. According to the herbarium and DNA barcoding data, *Ph. issikii* was first detected in Western Palearctic in the 1980s. Indeed, the earliest archival mine found in Europe dates back to 1987 in European Russia (Fig. 8). These results agree with the literature documenting *Ph. issikii* in Western Palearctic: the first finding of the species outside East Asia referred to Moscow and dated back to 1985^29^ (Fig. 8). Two years after, a foci of *Ph. issikii* was recorded in Voronezh, 500 km south-west of Moscow^28^ (see also Fig. 8), whereas in the next two decades it was recorded almost everywhere in European Russia^19^.

The dates of the first *Ph. issikii* records in the literature (1985 in Moscow) and those obtained in the present herbarium study (1987 in Zlatoust) are very similar (Fig. 8) but the distance between these two locations on the opposite parts of European Russia, i.e. in its western and eastern parts respectively, is considerable (about 1400 km). The findings of *Ph. issikii* in such a short time and in so distant locations may suggest that, by the time of the species detection, the moth had already spread across significant territory of European Russia that can also be the result of its multiple introductions from Eastern Palearctic and the host shift from East Asian limes to the European species of *Tilia* which turned out to be susceptible to the pest attacks^20^.

An unintentional introduction of *Ph. issikii* into European Russia could have happened after the Second World War when green landscaping in damaged cities and settlements required fast and effective restoration and greening programs^30,31^. At that period, botanical gardens were intensively created in European Russia, with numerous introductions of woody plant species from the East^32^. Thus, *Ph. issikii* may have escaped East Asia with its host plants, in particular *T. amurensis* and *T. mandshurica*, which have a native range in East Asia and are nowadays known in culture across most of European Russia^33^. However, we were unable to test this hypothesis because herbarium specimens were collected scarcely across Europe, European Russia and Siberia in the period of 1940–1970s.

Although we were unable to find earlier evidences of *Ph. issikii* invasion westwards despite extensive surveys of herbaria, our study clearly shows that in the West, the primary invasion foci occurred in European Russia, from where the moth started expanding westwards to other European countries and eastwards to Western Siberia (Fig. 8). These findings concur with the documentation of the moth’s expansion in these directions, as documented in the literature^19^. The timing of discovery of all those geographical records suggests that the invasion of *Ph. issikii* occurred westwards.

Finally, DNA barcode data allowed us to distinguish the archival larvae of *Ph. issikii* and species alike, in particular to assign the mines in herbarized lime specimens (*T. tomentosa* and *T. cordata* f. *vulgaris*) sampled in England (1915–1987), France (1927), and Italy (1942) to another leaf mining moth, *Phyllonorycter messaniella*. It is a polyphagous species with the native range in Europe and European Russia^34–36^. It develops on plants from several families, with oak as a main host (Fagaceae), in rare cases its mines can be found in Europe on limes, *Tilia* spp. (Malvaceae)^36,37^. Had we used morphology of the mines alone, we could have misdiagnosed this species as *Ph. issikii* and that would have significantly affected the dating of *Ph. issikii* invasion to Western Palearctic. In addition, our barcoding analysis showed no evidence of *Ph. issikii* introduction to the Nearctic in the past. Instead, we confirmed the presence of mines of two North American species in herbaria from Canada and the USA, namely *Ph. tiliacella* and *Ph. lucetiella*, previously known in these countries by literature^38^. Our study allowed to document the occurrence of these two species in several US states (see Fig. S3) where they were not previously known^36^.

Furthermore, DNA barcoding analysis of larvae remnants from the mines found in relatively recent herbaria from Primorsky Krai (the Russian Far East, sampling period 1995–1997) detected an unconfirmed candidate species (UCS) (see our study^22^ for details about the concept of UCS) feeding on *Tilia amurensis* and *T. taquetii*, the common host plants of *Ph. issikii*. In a previous paper, we discovered this *Tilia*-feeding UCS in the same geographical region and also in Japan through direct sampling of larvae, pupae and adults in nature^20^. This UCS may represent a cryptic species occurring in sympatry with *Ph. issikii* and sharing the same host plants. Despite being assigned to a different BIN with a minimum interspecific p-distance to *Ph. issikii* of 5.13%, morphological diagnostic characters, such as differences in male genitalia^20^, remain tentative.

To conclude, the survey of the large herbaria collected during the last 253 years in the northern hemisphere allowed us to clarify the invasion history of the lime leaf-mining micromoth, *Phyllonorycter issikii* and confirm its East Asian origin and the invasive status in the west of the Palearctic. The findings of the characteristic leaf mines in herbaria and molecular data recovered from larvae and pupae found in the mines in pressed leaves significantly improved our knowledge about the past range of the leaf miner in East Asia, and identified its trophic associations with East Asian limes. Moreover, our results highlighed the contribution of *Ph. issikii* populations from the Russian Far East, Japan and China to the invasion westwards of the Palearctic and confirmed its absence from the surveyed herbaria in the Nearctic. Finally, our study highlighted the importance of historical herbaria in studying past distribution and diversity of endophagous folivore organisms, including pestiferous and invasive species. As such, sampling and storing herbarium collection, including plant vouchers carrying any type of damage, and their wider use can be especially beneficial for invasive ecology research.

## Methods

### Studied herbarium collections

To reconstruct the past range and clarify the invasion history of *Ph. issikii*, we examined historical herbaria of *Tilia* spp. collected in the last 253 years (1764–2016) in the Palearctic, deposited in 20 museums and botanical gardens across Eurasia (Table S7). Additionally, the herbarium specimens collected in the last 109 years (1818–2008) in the Nearctic, where limes are also distributed^39^, were examined to check for *Ph. issikii* introduction in the past. So far, *Ph. issikii* has not been documented from the Nearctic^40^.

In total, we examined 15,692 herbarium specimens of *Tilia* of which 15,009 specimens (96%) originated from the Palearctic and 683 (4%) from the Nearctic (Fig. S4), altogether accounting for about 1.4 million lime leaves. The majority of herbarium specimens from the Palearctic (11,460, i.e. 73% of all studied specimens) came from the putative invaded range of *Ph. issikii* (i.e. from Europe, European Russia and Western Siberia). In Europe alone, the specimens originated from 30 different countries, altogether accounting for 8450 specimens (54%) (Fig. S4). Nearly in all these countries *Ph. issikii* was known by literature records^18–20,41^, except in the Scandinavian countries and England, where *P. issikii* has not been documented yet^36^. The other 3549 herbarium specimens (22.6% of all studied specimens) originated from the putative native range, East Asia, in particular from the RFE (2202 specimens, 14%), China (940 specimens, 6%), Japan (339 specimens, 2.2%), and Korea (68 specimens, 0.4%) (Fig. S4). *Ph. issikii* was historically known from these regions/countries, with the type locality in Japan^14–16^, except from China, where it was recorded for the first time in 2015 in Tianjin^20^.

In the Nearctic, 638 out of 683 herbarium specimens originated from North America, i.e. from 16 US states (604 specimens, i.e. 3.9% of all studies specimens) and from two Canadian provinces (34 specimens, i.e. 0.2%) (Fig. S4). The other 45 herbarium specimens (0.3%) originated from Northern Mexico (Fig. S4).

### Surveyed lime species

Given the ability of *Ph. issikii* to develop on different limes^14,20,41^, specimens of all representatives of the genus *Tilia* stored in herbaria were examined. Altogether they accounted for 117 lime species and more than 80 hybrids across the Palearctic and Nearctic (Table S8). Bearing in mind that the taxonomy of limes has been revised several times^42,43^, some species indicated on the labels of herbarium specimens have been synonymized by botanists since the collection date. However, in order to keep the historical record and avoid misinterpretation of the primary identifications, we refer to the lime species names as they were originally stated on the vouchers.

### Examination of herbarium specimens

**Figure 9:**
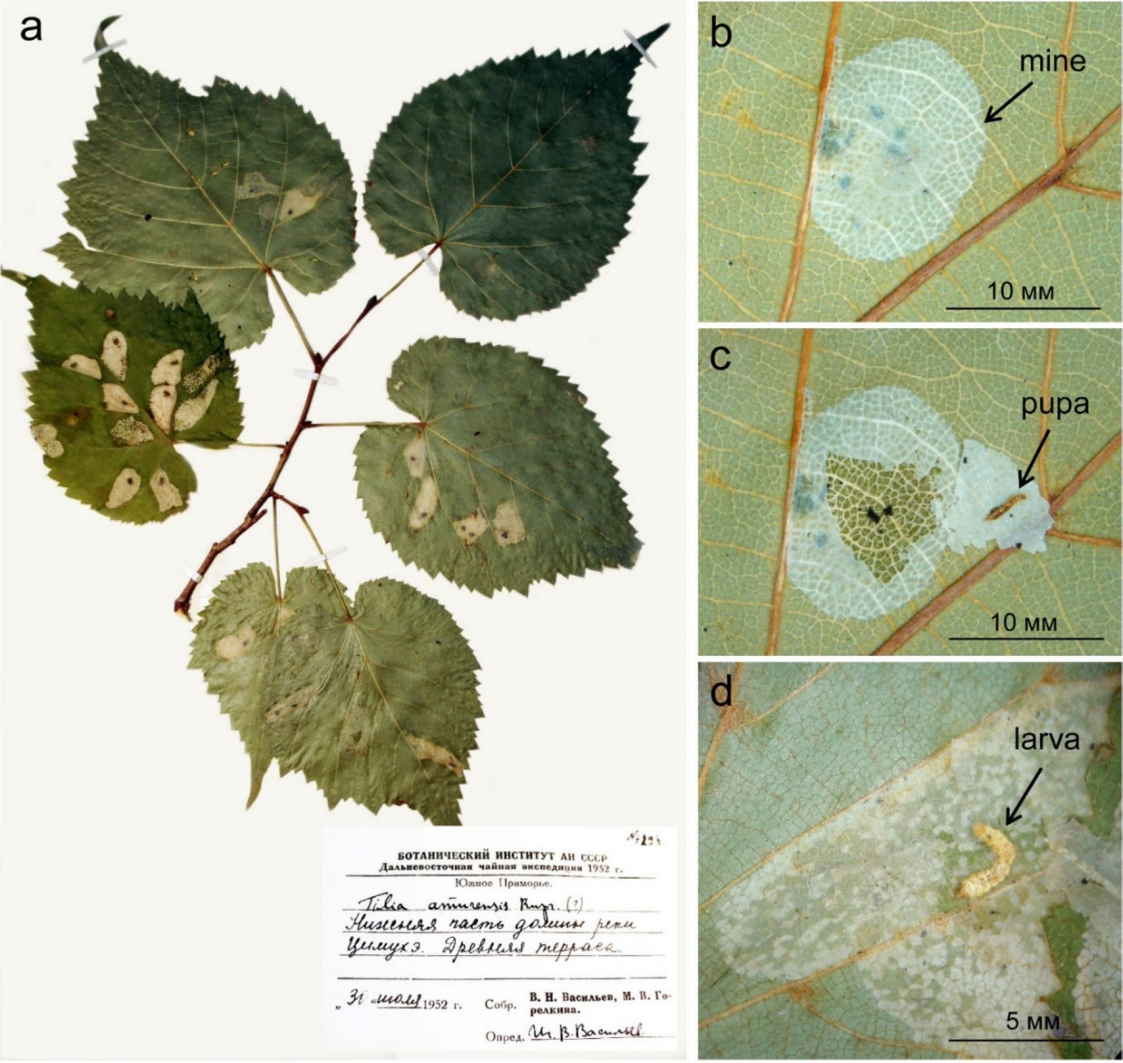
An example of herbarium specimen with *Phyllonorycter issikii* mines (**a**), the mine (**b**), opened mine with the pupa (**c**), opened mine with the larva (**d**). The data from the label at the herbarium specimen originating from the Russian Far East: № 296, Botanical institute of the Academy of Sciences of USSR, Far Eastern Tea Expedition of the year 1952; South Primorye, *Tilia amurensis* Rupr., the lower part the valley of the river Tsimukhe, ancient terrace; 31.VII.1952 coll; V. N. Vasilyev, M. V. Gorelkina coll.; I. V. Vasilyev det. Photographs taken by N. Kirichenko.

The herbarium specimens were carefully examined for the presence of the typical *Phyllonorycter* mines, i.e. whitish oval flat or tentiform blotches on leaf lamina (Fig. 9). Only the mines of a diameter 3–5 mm onward were taken into consideration (by the end of development, the diameter of *Ph. issikii* mines may reach 15 mm). The mines of a smaller size are, in general, difficult to spot on pressed leaves and to identify reliably.

The pressed leaves were examined from the lower side where *Ph. issikii* lay eggs and make blotch mines^14,18^. The total number of leaves in a herbarium specimen, the number of leaves with the mines, and the number of mines on the leaves were counted.

Randomly selected mines were opened to sample late instar larvae or pupae for further analyses (Fig. 9). The mines were opened by making an incision in the epidermis in the area where larvae or pupae were spotted (Fig. 9). To minimize contamination, the forceps used for picking insects were thoroughly rinsed with 95% ethanol solution after each manipulation. The sampled larvae and pupae (93 individuals) were placed individually into 2 ml microtubes with hermetically sealed lids (Axygen, USA), labeled and stored in a freezer at − 20 °C prior for morphologic (pupae) and molecular genetic (larvae and pupae) analyses. Additionally, 17 pupal exuvia were sampled for morphological identification.

### Morphological diagnostics

Pupae and pupal exuvia were examined at magnification × 20–40 using ZEISS Stemi DV4 binocular (Germany); pupal cremaster was studied under the magnification × 60 using the microscope Olympus CX21 (Japan). Based on the mine characters (i.e. the position of mines on the leaf surface, presence/absence of folds on epidermis covering mine, frass pattern) and the sculpture of pupal cremaster^37,38,44^, the mines were attributed to lime-feeding *Phyllonorycter* species known in the Palearctic and Nearctic (Table S9). The typical oval mines, mostly situated between the secondary veins on the lower epidermis, with no folds on epidermis covering mine^37^, inside which neither larvae nor pupae were found from the localities where *Ph. issikii* is known in the Palearctic were attributed to the latter*.*

### Molecular genetic analysis

Archival larvae and pupae dissected from the mines on pressed leaves (overall 93 specimens) were analyzed using DNA barcoding paired with third generation sequencing to confirm species identity, determine historic haplotypes, overview past range, find early evidences of *Ph. issikii* invasion in the Palearctic, and check for the species presence in the Nearctic back in time. Nondestructive approach was used to save the vouchers. In the archive insects, up to 11 overlapping fragments (ranging from 150 to 230 nucleotides) of the mtDNA COI gene region (658 bp) were sequenced. DNA extraction, amplification, and sequencing were performed at the Canadian Center for DNA-barcoding at the University of Guelph (Canada). The molecular protocols from a previous study^23^ were adapted for single molecular real-time sequencing (SMRT) on Sequel platform (Pacific Biosciences)^25^. For the assembly of nucleotide contigs, multiple fragments were aligned to a reference sequence^25^ and visually inspected using the Codon Code Aligner V.9.0.1 software (CodonCode Corporation)

### Data analysis

The Spearman's rank correlation (R) was used to assess sequencing success, i.e. the relationship between the length of the sequenced fragment (maximal of 658 bp) and the age of the archival specimens, (*p* < 0.05). In further analyses only sequences with the length of at least 300 bp were used; shorter sequences with missing diagnostic sites were excluded. Archival specimens were identified by their DNA barcodes to the species level using the Barcode of Life Database (BOLD, http://www.boldsystems.org) identification engine^45^.

The phylogenetic tree analyzing the relatedness of the archival specimens was built using the Maximum likelihood estimation algorithm, the Kimura's two-parameter (K2P) model and the bootstrap with 2500 iterations. Thus, all genetic distances in the study are K2P unless stated otherwise. In the phylogenetic tree, the topology of the basal branches was considered robust at a value ≥ 70. Phylogenetic analysis was run in Mega X^46^. The haplotype diversity was computed as follows, H = (N/(n − 1)) × (1 − ∑x_i_^2^), where x_i_ is the relative haplotype frequency of each haplotype in the sample and N is the sample size^47^.

To determine the haplotypes, the sequences of the archival *Ph. issikii* specimens were analyzed in the total alignment with the pool of the DNA barcodes previously accumulated in the study of 377 *Ph. issikii* individuals in the modern species range in the Palearctic, https://doi.org/10.5883/DS-TILIAPHY^20^. For sequences with different lengths, only the length aligned with compared sequences was taken into the consideration for distance calculation and haplotype determination. The haplotypes identified in archival specimens that were identical to those occurring in the modern *Ph. issikii* range, as determined in our previous study^20^, were assigned the same haplotype name (for example, H2—“Haplotype 2”, common for modern and historical ranges) and shaded by the same color on the haplotype network. The haplotypes identified in the archival specimens that differed from those known in the *Ph. issikii* modern range were considered as “old” haplotypes, i.e. present in the past but absent in the moth’s modern range. The median-joining haplotype network was constructed in TCS 1.21 implementing a statistical parsimony algorithm^48^.

The geographic distribution of COI haplotypes was mapped using ArcGIS 9.3^49^. The historical range of *Phyllonorycter* species was illustrated in accordance with the finding time scale (in a 10-year interval). The *Ph. issikii* distribution scenario in Western Palearctic was generated based on the dating records of the first occurrence of *Ph. issikii* in different locations as per published data^18,19,50^ and the herbarium data analyses performed in the present study.

In the Palearctic, the number of herbarium specimens carrying *Phyllonorycter* mines was analyzed for the putative native range (East Asia), that we referred to as Eastern Palearctic, versus the putative invaded range (European countries, European Russian and Western Siberia), that we referred to as Western Palearctic. The average number of leaves with the mines per herbarium specimen was calculated and compared between these two macroregions. We also compare the East and the West by the dating of the mines in historical herbaria (i.e. distribution of records across time scale). The Mann–Whitney nonparametric U-test, allowing analyzing small and unequal sampling sets, was used for data comparison (STATISTICA 12.6 software, Stat Soft. Inc., USA).

### Ethics approval

The herbaria surveys were carried out in respect to the Institutional and International ethical guidelines. The protocol of archival larvae and pupae sampling from herbarized leaves was approved by the herbarium curators in all visited herbarium depositaria.

## Supplementary Information

Supplementary Information.

## Data Availability

Seventy one newly generated sequences, along with the voucher data, images, and other supporting information are deposited in BOLD; the sequences have been also deposited in GenBank. All data (including GenBank accession numbers) are available from BOLD in the dataset DS-HERBPHYL (https://doi.org/10.5883/DS-HERBPHYL).
