# Supplementary material for: Tracing the invasion of a leaf-mining moth in the Palearctic through DNA barcoding of historical herbaria
Source: Sci Rep. 2022 Mar 24;12:5065. doi: 10.1038/s41598-022-08894-7 (PMC8948198; doi:10.1038/s41598-022-08894-7)
Supplement: Supplementary file 1 — Supplementary Information. [file 41598_2022_8894_MOESM1_ESM.pdf]

SUPPLEMENTARY

TITLE: Tracing the invasion of a leaf-mining moth in the Palearctic through DNA barcoding of historical herbaria

AUTHORS: Natalia I. Kirichenko, Evgeny V. Zakharov, Carlos Lopez-Vaamonde

Table S1. Presence of *Phyllonorycter messaniella* mines in herbarium specimens of three lime species in Western Europe in 1915–1942*.

№	Country	Region (city)	Number of				Mined leaves per herb. specimen, %	Year	*Tilia* species**
			herb. speci mens	mines	leaves with mines	leaves in herb. speci-men			
1	England	Hampshire	1	1	1	10	10	1915	*T. petiolaris*
2	England	Gloucestershire	1	3	3	12	25	1927	*T. tomentosa*
3	England	Gloucestershire	1	1	1	8	13	1927	*T. tomentosa*
4	Italy	Campania (Portichi)	1	3	1	2	50	1927	*T.* × *vulgaris*
5	France	Normandy (Caen)	1	1	1	2	50	1942	*T. tomentosa*
6	France	Normandy (Caen, Venoix)	1	2	1	1	100	1942	*T. tomentosa*
		**In total:**	**6**	**11**	**8**	**35**	**Average: 41±14%**	**—**	**—**

*The species identified by mine morphology; where possible, by pupal morphology and by DNA barcoding of larvae and pupae dissected from the mines. All herbarium specimens with *Ph. messaniella* mines are stored at the Natural History Museum (London). ***T.* × *vulgaris* is presently considered a junior synonym of *T.* × *europaea* (which is a hybrid of *T. cordata* and *T. platyphyllos*). — not applicable.

Table S2. Presence of *Phyllonorycter lucetiella* and *Ph. tiliacella* mines in the herbarium specimens of different limes, *Tilia* spp., in North America in 1824–2010.

№	Count-ry	Province or state	Number of				Mined leaves per herb. specimen, %	Year	Lime species*
			herb. speci-mens	mines	leaves with mines	leaves in herb. specimen			
Phyllonorycter lucetiella									
1	Canada	Ontario	1	1	1	5	20	1983	*T. americana*
2	Canada	Ontario	1	1	1	9	11	1962	*T. americana*
3	Canada	Quebec	1	1	1	8	13	1941	*T. glabra*
4	USA	Alabama	1	1	1	5	20	2008	*T. caroliniana*
5	USA	Arkansas	1	1	1	8	13	1972	*Tilia* sp.
6	USA	Virginia	1	1	1	6	17	1994	*T. caroliniana*
7	USA	Illinois	1	8	8	8	100	1979	*T. americana*
8	USA	Mississippi	1	3	3	14	21	2000	*T. americana*
9	USA	Mississippi	1	1	1	10	10	2000	*T. americana*
10	USA	Mississippi	1	4	4	15	27	2000	*T. americana*
11	USA	Mississippi	1	1	1	6	17	2000	*T. americana*
12	USA	Missouri	1	2	2	8	25	1850	*Tilia* sp.
13	USA	Missouri	1	3	3	12	25	1922	*T. americana*
14	USA	Missouri	1	5	5	5	100	1921	*T. americana*
15	USA	Missouri	1	1	1	3	33	1921	*T. americana*
16	USA	Missouri	1	1	1	7	14	1988	*T. americana*
17	USA	Michigan	1	1	1	11	9	1994	*T. americana*
18	USA	New York	1	1	1	6	17	1994	*T. americana*
19	USA	New York	1	1	1	10	10	1948	*T.heterophylla*
20	USA	New York	1	1	1	9	11	2010	*T. americana*
21	USA	New York	1	1	1	12	8	1980	*Tilia* sp.
22	USA	New York	1	2	2	13	15	1980	*Tilia* sp.
23	USA	New York	1	3	3	16	19	1899	*T. americana*
24	USA	N. Carolina	1	2	2	5	40	1994	*T. caroliniana*
25	USA	N. Carolina	1	4	2	11	18	1994	*T. caroliniana*
26	USA	N. Carolina	1	8	5	8	63	1994	*T. caroliniana*
27	USA	N. Carolina	1	1	1	3	33	1994	*T. caroliniana*
28	USA	N. Carolina	1	7	5	10	50	1994	*T. caroliniana*
29	USA	N. Carolina	1	2	2	5	40	1994	*T. caroliniana*
30	USA	N. Carolina	1	10	10	11	91	1994	*T. caroliniana*
31	USA	N. Carolina	1	1	1	3	33	1994	*T. caroliniana*
32	USA	Texas	1	1	1	5	20	1872	*T. caroliniana*
33	USA	Florida	1	2	2	7	29	2000	*T. caroliniana*
34	USA	Florida	1	1	1	7	14	2000	*T. caroliniana*
		**In total:**	**34**	**84**	**77**	**281**	**Average: 29±5%**	**—**	**—**

№	Count-ry	Province or state	Number of				Mined leaves per herb. specimen, %	Year	Lime species*
			herb. speci-mens	mines	leaves with mines	leaves in herb. specimen			
				Phyllonorycter tilicella					
35	USA	Ohio	1	2	2	3	67	1905	*Tilia* sp.
36	USA	Ohio	1	1	1	5	20	1924	*T.heterophylla*
37	USA	Pennsylva-nia	1	1	1	7	14	1824	*T. americana*
		**In total:**	**3**	**4**	**4**	**15**	**Average: 34±17%**	**—**	**—**

*The limes *T. glabra, T. caroliniana, T. heterophylla* are presently considered either as a synonyms or subspecies of *Tilia americana*. — not applicable.

Table S3. Presence of *Phyllonorycter issikii* mines in the herbarium specimens of different lime species in the Palearctic in the last 253 years (1764–2016).

**№**	**Lime species**	**Number of herbarium specimens with mines**		**Number of mines**		**Number of leaves with mines**	**Number of leaves in herbarium specimen**	**Number of mined leaves per herb. specimen, %**
		**absolute value**	**% from all number of herbarium specimens with mines**	**absolute value**	**% from all mines**			
**(a) Western Palearctic (putative invaded range)***								
1	*Tilia cordata*	15	7	102	7,9	62	300	21
2	*T. platyphyllos*	7	3	32	2,5	20	57	35
**(a) In total:**		**22**	—	**134**	—	**82**	**357**	—
**(b) Eastern Palearctic (putative native range)****								
3	*T. amurensis*	74	33	661	51,3	327	1647	20
4	*T. taquetii*	42	19	312	24,2	197	1016	19
5	*T. mandshurica*	24	11	36	2,8	34	171	20
6	*T. japonica*	11	5	15	1,2	14	235	6
7	*T. chinensis*	8	4	11	0,9	11	135	8
8	*T. maximowicziana*	7	3	13	1,0	8	91	9
9	*T. tuan*	5	2	10	0,8	8	118	7
10	*T. mongolica*	4	2	5	0,4	5	101	5
11	*T. laetevirens*	3	1	26	2,0	20	28	71
12	*T. kiusiana*	2	1	5	0,4	5	31	16
13	*T. pekinensis*	1	0	3	0,2	2	6	33
14	*T. intonsa*	1	0	2	0,2	2	9	22
15	*T. leptocarya*	1	0	1	0,1	1	7	14
16	*T. rufa*	1	0	3	0,2	3	25	12
17	*T. miqueliana*	1	0	1	0,1	1	12	8
18	*T. koreana*	1	0	1	0,1	1	15	7
19	*T. paucicostata*	1	0	1	0,1	1	19	5
20	*T. diviticata*	1	0	1	0,1	1	25	4
21	*Tilia. sp*	15	7	47	3,6	34	338	10
**(b) In total:**		**203**	—	**1154**	—	**675**	**4029**	—
**(a+b) In total:**		**225**	—	**1288**	—	**757**	**4386**	—

*In Western Palearctic, following regions and countries were studied: Europe, Russia (European part of Russia, Western Siberia). **In Eastern Palearctic: the Russian Far East, Korea, China, and Japan. — not applicable.

Table S4. Specimen data of 71 DNA barcoded archival larvae and pupae of *Tilia*-feeding *Phyllonorycter* dissected from the mines in herbaria (dated between 1859–2014) in the Northern Hemisphere and 11 DNA barcodes of specimens collected in relatively recent time in nature borrowed from BOLD for comparison. The dataset is available at: dx.doi.org/10.5883/DS-HERBPHYL.

№	*Phyllonorycter* species* and development stage in []**	Process ID	GenBank accession ***	Collector	Host plant****	Country	Locality*****	Date of collection	Latitude	Longi-tude	Elevation, m
**Archival specimens of lime-feeding *Phyllonorycter* spp. dissected from herbarium**											
1	*Ph. issikii* [L]	LMINH143-19	OM311966	N. Kirichenko	*T. cordata*	Germany	Hessen	16.VII.2014	50.622	9.088	381
2	*Ph. issikii* [L]	LMINH144-19	OM311967	N. Kirichenko	*T. cordata*	Germany	Hessen	16.VII.2014	50.622	9.088	381
3	*Ph. issikii* [L]	LMINH052-19	OM311992	N. Kirichenko	*T. platyphyllos*	Italy	Veneto	04.X.2011	46.004	12.000	495
4	*Ph. issikii* [P]	LMINH020-19	OM312016	N. Kirichenko	*T. amurensis*	China	Jilin	31.VIII.1951	43.111	126.301	396
5	*Ph. issikii* [P]	LMINH022-19	OM312008	N. Kirichenko	*T. mandshurica*	China	Shandong	06.VIII.1924	35.665	117.710	201
6	*Ph. issikii* [L]	LMINH023-19	OM312018	N. Kirichenko	*T. taquetii*	China	Heilongjiang	20.VI.1903	46.021	127.567	188
7	*Ph. issikii* [L]	LMINH026-19	OM311960	N. Kirichenko	*Tilia. sp.*	China	Heilongjiang	26.VI.1903	46.146	129.178	439
8	*Ph. issikii* [L]	LMINH030-19	OM311957	N. Kirichenko	*T. amurensis*	China	Jilin	29.VI.1896	44.159	123.852	167
9	*Ph. issikii* [L]	LMINH031-19	OM312017	N. Kirichenko	*T. amurensis*	China	Jilin	29.VI.1896	44.159	123.852	167
10	*Ph. issikii* [L]	LMINH032-19	N/A	N. Kirichenko	*T. amurensis*	China	Jilin	29.VI.1896	44.159	123.852	167
11	*Ph. issikii* [L]	LMINH036-19	N/A	N. Kirichenko	*T. laetevirens*	China	Gansu	01.VI.1911	35.829	103.811	2435
12	*Ph. issikii* [L]	LMINH038-19	N/A	N. Kirichenko	*T. mandshurica*	China	Jilin	01.VII.1896	43.055	127.392	543
13	*Ph. issikii* [P]	LMINH001-19	OM311998	N. Kirichenko	*T. cordata*	Russia	KO	19.VIII.2014	57.906	41.253	153
14	*Ph. issikii* [L]	LMINH002-19	OM311970	N. Kirichenko	*T. cordata*	Russia	KO	27.VIII.2014	58.294	42.402	146
15	*Ph. issikii* [P]	LMINH003-19	OM311988	N. Kirichenko	*T. cordata*	Russia	ChO	28.VII.1987	55.159	59.615	601
16	*Ph. issikii* [L]	LMINH004-19	OM311983	N. Kirichenko	*T. cordata*	Russia	SO	29.VIII.1990	53.433	49.672	98
17	*Ph. issikii* [L]	LMINH006-19	OM312005	N. Kirichenko	*T. amurensis*	Russia	PK	17.VII.1951	43.579	131.992	63
18	*Ph. issikii* [P]	LMINH007-19	OM312007	N. Kirichenko	*T. amurensis*	Russia	PK	17.VII.1951	43.579	131.992	63
19	*Ph. issikii* [P]	LMINH008-19	OM312003	N. Kirichenko	*T. amurensis*	Russia	PK	17.VII.1951	43.579	131.992	63
20	*Ph. issikii* [L]	LMINH009-19	OM311963	N. Kirichenko	*T. amurensis*	Russia	PK	30.VII.1952	43.315	132.517	237
21	*Ph. issikii* [L]	LMINH010-19	OM311971	N. Kirichenko	*T. amurensis*	Russia	PK	25.VII.1952	43.654	132.524	211

№	*Phyllonorycter* species* and development stage in []**	Process ID	GenBank accession ***	Collector	Host plant****	Country	Locality*****	Date of collection	Latitude	Longi-tude	Elevation, m
22	*Ph. issikii* [L]	LMINH011-19	OM311974	N. Kirichenko	*T. amurensis*	Russia	PK	26.VII.1950	43.669	132.527	452
23	*Ph. issikii* [L]	LMINH012-19	OM311968	N. Kirichenko	*T. amurensis*	Russia	AO	02.VIII.1914	54.636	126.748	509
24	*Ph. issikii*[L]	LMINH013-19	OM311997	N. Kirichenko	*T. amurensis*	Russia	PK	24.VII.1936	43.699	132.169	236
25	*Ph. issikii* [L]	LMINH014-19	OM311959	N. Kirichenko	*T. taquetii*	Russia	PK	14.VII.1951	43.696	132.167	211
26	*Ph. issikii* [L]	LMINH015-19	OM312012	N. Kirichenko	*T. taquetii*	Russia	PK	14.VII.1951	43.696	132.167	211
27	*Ph. issikii* [L]	LMINH016-19	OM311956	N. Kirichenko	*T. taquetii*	Russia	PK	17.VII.1936	43.669	132.527	452
28	*Ph. issikii* [L]	LMINH017-19	OM312015	N. Kirichenko	*T. taquetii*	Russia	PK	21.VII.1936	43.669	132.527	452
29	*Ph. issikii* [P]	LMINH018-19	OM311989	N. Kirichenko	*T. taquetii*	Russia	PK	28.VII.1936	43.669	132.527	452
30	*Ph. issikii* [P]	LMINH019-19	OM312010	N. Kirichenko	*T. taquetii*	Russia	PK	28.VII.1936	43.669	132.527	452
31	*Ph. issikii* [L]	LMINH041-19	OM311978	N. Kirichenko	*T. taquetii*	Russia	PK	01.VI.1981	43.854	135.152	179
32	*Ph. issikii* [L]	LMINH042-19	OM311976	N. Kirichenko	*T. taquetii*	Russia	PK	01.VI.1936	43.669	132.527	452
33	*Ph. issikii* [P]	LMINH046-19	OM311953	N. Kirichenko	*T. amurensis*	Russia	PK	01.VI.1936	43.669	132.527	452
34	*Ph. issikii* [L]	LMINH047-19	OM312011	N. Kirichenko	*T. amurensis*	Russia	PK	01.VI.1941	45.734	135.156	439
35	*Ph. issikii* [L]	LMINH055-19	OM311994	N. Kirichenko	*T. taquetii*	Russia	PK	01.VII.1951	43.579	131.992	63
36	*Ph. issikii* [L]	LMINH098-19	OM312001	N. Kirichenko	*T. mandshurica*	Russia	PK	16.VII.1968	43.316	132.684	267
37	*Ph. issikii* [L]	LMINH103-19	OM311969	N. Kirichenko	*T. amurensis*	Russia	PK	24.VI.1987	43.177	131.970	184
38	*Ph. issikii* [L]	LMINH104-19	OM311973	N. Kirichenko	*T. amurensis*	Russia	PK	04.VII.1998	43.692	132.155	147
39	*Ph. issikii* [L]	LMINH105-19	OM311991	N. Kirichenko	*T. amurensis*	Russia	PK	17.VII.1992	43.197	132.111	8
40	*Ph. issikii* [P]	LMINH106-19	OM312004	N. Kirichenko	*T. amurensis*	Russia	PK	17.VII.1992	43.197	132.111	8
41	*Ph. issikii* [P]	LMINH107-19	OM311977	N. Kirichenko	*T. amurensis*	Russia	KhK	25.VIII.1985	50.345	137.719	92
42	*Ph. issikii* [L]	LMINH108-19	OM311979	N. Kirichenko	*T. amurensis*	Russia	KhK	01.VI.1961	49.991	135.304	269
43	*Ph. issikii* [L]	LMINH109-19	OM311985	N. Kirichenko	*T. amurensis*	Russia	KhK	01.VI.1961	49.991	135.304	269
44	*Ph. issikii* [L]	LMINH110-19	OM311962	N. Kirichenko	*T. amurensis*	Russia	KhK	01.VI.1961	49.991	135.304	269
45	*Ph. issikii* [L]	LMINH111-19	OM311986	N. Kirichenko	*T. amurensis*	Russia	KhK	06.VIII.1977	54.164	126.157	851
46	*Ph. issikii* [P]	LMINH113-19	OM312013	N. Kirichenko	*T. heterophylla*	Russia	PK	23.VIII.1975	42.483	130.750	3
47	*Ph. issikii* [P]	LMINH119-19	OM311984	N. Kirichenko	*T. heterophylla*	Russia	AO	23.VII.1859	54.021	126.743	686
48	*Ph. issikii* [L]	LMINH147-19	OM312014	N. Kirichenko	*T. taquetii*	Russia	PK	18.VII.1951	43.634	132.488	343

№	*Phyllonorycter* species* and development stage in []**	Process ID	GenBank accession ***	Collector	Host plant****	Country	Locality*****	Date of collection	Latitude	Longi-tude	Elevation, m
49	*Ph. issikii* [L]	LMINH039-19	N/A	N. Kirichenko	*T. maximowicziana*	Japan	Hokkaido	01.VI.1905	43.349	141.639	171
50	*Ph. issikii* [L]	LMINH123-19	OM311981	N. Kirichenko	*T. maximowicziana*	Japan	Hokkaido	19.VII.1956	42.927	141.125	598
51	*Ph. messaniella* [L]	LMINH121-19	OM311975	N. Kirichenko	*T. vulgaris*	Italy	Campania, Portichi	12.V.1927	40.812	14.345	59
52	*Ph. messaniella* [P]	LMINH122-19	OM311965	N. Kirichenko	*T. tomentosa*	France	Normandia, Caen	12.IX.1942	49.163	-0.370	10
53	*Ph. tiliacella* [P]	LMINH120-19	OM311982	N. Kirichenko	*T. heterophylla*	USA	Ohaio	01.VI.1960	40.616	-82.879	320
54	*Ph. tiliacella* [P]	LMINH153-19	OM311980	N. Kirichenko	*T. americana*	USA	Pennsylvania	01.VIII.1894	39.843	-77.960	334
55	*Ph. lucetiella* [L]	LMINH040-19	OM311995	N. Kirichenko	*T. glabra*	Canada	Ontario	01.VI.1941	45.444	-75.611	84
56	*Ph. lucetiella* [L]	LMINH043-19	OM311958	N. Kirichenko	*T. americana*	USA	Massachusetts	17.IX.1979	42.499	-71.546	42
57	*Ph. lucetiella* [L]	LMINH044-19	OM311987	N. Kirichenko	*T. americana*	USA	Massachusetts	17.IX.1979	42.499	-71.546	42
58	*Ph. lucetiella* [L]	LMINH045-19	OM311990	N. Kirichenko	*T. americana*	USA	Massachusetts	17.IX.1979	42.499	-71.546	42
59	*Ph. lucetiella* [L]	LMINH145-19	OM311972	N. Kirichenko	*T. heterophylla*	USA	New York	15.VII.1948	42.742	-76.597	309
60	*Ph. lucetiella* [L]	LMINH152-19	N/A	N. Kirichenko	*T. americana*	USA	New York	01.VI.2010	40.731	-73.991	5
61	*Ph. lucetiella* [L]	LMINH128-19	OM311961	N. Kirichenko	*T. caroliniana*	USA	N.Carolina	08.X.1994	34.540	-77.397	1
62	*Ph. lucetiella* [L]	LMINH129-19	OM311999	N. Kirichenko	*T. caroliniana*	USA	N.Carolina	08.X.1994	34.540	-77.397	1
63	*Ph. lucetiella* [L]	LMINH130-19	OM311955	N. Kirichenko	*T. caroliniana*	USA	N.Carolina	08.X.1994	34.540	-77.397	1
64	*Ph. lucetiella* [L]	LMINH131-19	OM311954	N. Kirichenko	*T. caroliniana*	USA	N.Carolina	08.X.1994	34.540	-77.397	1
65	*Ph. lucetiella* [L]	LMINH132-19	OM312002	N. Kirichenko	*T. caroliniana*	USA	N.Carolina	08.X.1994	34.540	-77.397	1
66	*Ph. lucetiella* [L]	LMINH133-19	OM311993	N. Kirichenko	*T. caroliniana*	USA	Florida	11.VIII.2000	30.521	-84.760	38
67	*Ph. lucetiella* [P]	LMINH137-19	OM311996	N. Kirichenko	*T. caroliniana*	USA	Florida	07.VIII.2000	30.575	-85.085	36
68	*Phyllonorycter* sp. [L]	LMINH096-19	OM312006	N. Kirichenko	*T. amurensis*	Russia	PK	01.VIII.1995	42.765	132.335	256
69	*Phyllonorycter* sp. [L]	LMINH099-19	OM311964	N. Kirichenko	*T. taquetii*	Russia	PK	03.X.1997	43.006	131.848	127
70	*Phyllonorycter* sp. [P]	LMINH101-19	OM312000	N. Kirichenko	*T. taquetii*	Russia	PK	03.X.1997	43.006	131.848	127
71	*Phyllonorycter* sp. [L]	LMINH102-19	OM312009	N. Kirichenko	*T. taquetii*	Russia	PK	24.VI.1987	43.417	133.485	752

DNA barcodes of specimens collected in nature in modern time

№	*Phyllonorycter* species* and development stage in []**	Process ID	GenBank accession ***	Collector	Host plant****	Country	Locality*****	Date of collection	Latitude	Longi-tude	Elevation, m
1	*Ph. issikii* [I]	PHLAD034-11	JN280415	P. Huemer	—	Италия	South Tyrol	14.IX.2011	46.428	11.300	643
2	*Ph. issikii* [I]	ISSIK001-12	KX818414	N. Kirichenko	*T. cordata*	Russia	MO	21.VI.2010	54.839	37.604	162
3	*Ph. issikii* [L]	ISSIK269-14	KX818601	N. Kirichenko	*T. taquetii*	Russia	PK	28.VIII.2011	43.689	132.157	160
4	*Ph. issikii* [L]	ISSIK378-15	KX818449	N. Kirichenko	*T. mandshurica*	Russia	PK	17.VII.2013	43.681	132.160	224
5	*Ph. issikii* [I]	GRAAM048-13	KX818530	G. Deschka	*T. maximowicziana*	Japan	Hokkaido	28.IX.1966	43.113	140.453	30
6	*Ph. issikii* [I]	ISSIK297-14	KX818411	K. Tokashi	*T. maximowicziana*	Japan	Hokkaido	13.IX.2014	43.034	141.315	121
7	*Ph. issikii* [I]	ISSIK317-14	KX818397	K. Tokashi	*T. maximowicziana*	Japan	Hokkaido	13.IX.2014	43.034	141.315	121
8	*Ph. messaniella* [I]	GRPAL161-11	JN280183	A. Cama	—	France	Loire	12.XII|.2006	47.250	0.210	33
9	*Ph. lucetiella* [L]	MICRU060-15	KX818687	J.-F. Landry	*T. americana*	Canada	Quebec	27.IX.2015	45.469	-75.811	180
10	*Phyllonorycter* sp. [L]	ISSIK371-14	KX818698	K. Tokashi	*T. japonica*	Japan	Honshu	01.IX.2014	38.260	140.850	244
						**Outgroup**					
11	*Tischeria* sp. [L]	ISSIK102-14	MK403710	N. Kirichenko	*T. taquetii*	Russia	PK	25.VIII.2011	43.689	132.157	160

*Species: sp. – putative new species. **Stage: L – larva, P – pupa, I – imago. ***Genbank accession: N/A – the number is not available for too short sequences (133 b.p.). ****Host plant: — no data available. *****Localities: SO – Sverdlovsk Oblast, KO – Kostroma Oblast, ChO – Chelyabinsk Oblast, AO – Amur Oblast, PK – Primorskiy Krai, KhK – Khabarovsk Krai.

Table S5. Geographical distribution, length of DNA sequence and year of collection of nine haplotypes of *Phyllonorycter issikii* (COI gene mtDNA) obtained from archival samples and present among modern specimens in the Palearctic*.

Haplo-type number	Country, region according to herbarium data**	Years	Obtained sequence length, bp.	Process ID	Presence of the haplotype in the modern *Ph. issikii* range***
H1	Russia, RFE	1951	563	LMINH006-19	Austria, Bulgaria, Hungary, Germany, Netherlands, Poland, Russia (European part), Slovenia, Ukraine, Finland, Czech Republic, Japan (Hokkaido)
	Russia, RFE	1952	563	LMINH009-19	
	Russia, RFE	1950	563	LMINH011-19	
	Russia, RFE	1936	563	LMINH013-19	
	Russia, RFE	1951	563	LMINH015-19	
	Russia, RFE	1936	563	LMINH016-19	
	Japan, Hokkaido	1956	563	LMINH123-19	
	Russia, ER	1987	563	LMINH003-19	
	Germany	2014	563	LMINH143-19	
	Germany	2014	563	LMINH144-19	
H2	Russia, RFE	1981	563	LMINH041-19	Russia (Siberia)
H8	Russia, RFE	1936	563	LMINH018-19	Austria, Bulgaria, Hungary, Germany, Italy, Lithuania, Russia (European part, Siberia, RFE), Slovenia, Ukraine, Finland
	Italy	2011	563	LMINH052-19	
H13	Russia, RFE	1951	563	LMINH008-19	Russia (Siberia)
	Russia, RFE	1992	563	LMINH105-19	
H22	China, Heilongjiang	1903	278	LMINH023-19	Russia (European part)
H23	Russia, RFE	1951	563	LMINH014-19	Austria, Bulgaria, Hungary, Germany, Lithuania, Netherlands, Poland, Russia (European part, Siberia), Ukraine, Finland
	Russia, RFE	1992	563	LMIMH106-19	
	Russia, RFE	1951	563	LMINH147-19	
	Russia, RFE	1961	563	LMINH108-19	
	Russia, ER	2014	563	LMINH001-19	
	Russia, ER	1990	563	LMINH004-19	
H26	China, Jilin	1951	563	LMINH020-19	Russia (RFE)
H28	Russia, RFE	1975	513	LMINH113-19	Russia (RFE)
H30	Russia, RFE	1998	563	LMINH104-19	Russia (RFE)

*Presence of the haplotype in the modern *Ph. issikii* range is given according to the previous study[20]. **RFE – Russian Far East, ER – European Russia. ***Countries and regions where the same haplotypes have been found in the historical and modern areas are underlined. The haplotypes in lines colored in yellow correspond to the Cluster B, the remaining represent the Cluster A (as shown in Figure 7).

Table S6. The geographical distribution of 23 unique historical haplotypes of *Phyllonorycter issikii* (COI gene mtDNA) found in past exclusively in East Asia.

Haplotype number	Country, region*	Years	Obtained sequence length, bp	Lime species	Process ID
U1	Russia, RFE (PK)	1951	563	*T. maximowicziana*	LMINH007-19
U2	Russia, RFE (KhK)	1985	563	*T. amurensis*	LMINH107-19
U3	Russia, RFE (PK)	1952	379	*T. amurensis*	LMINH010-19
U4	Russia, RFE (PK)	1936	511	*T. taquetii*	LMINH019-19
U5	Russia, RFE (PK)	1951	513	*T. taquetii*	LMINH055-19
U6	Russia, RFE (PK)	1936	408	*T. amurensis*	LMINH046-19
U7	Russia, RFE (KhK)	1961	458	*T. amurensis*	LMINH109-19
U8	Russia, RFE (KhK)	1977	563	*T. amurensis*	LMINH111-19
U9	Russia, RFE (AO)	1914	563	*T. amurensis*	LMINH012-19
U10	Russia, RFE (KhK)	1961	563	*T. amurensis*	LMINH110-19
U11	Russia, RFE (AO)	1859	408	*T. amurensis*	LMINH119-19
U12	Japan, Hokkaido	1905	133	*T. maximowicziana*	LMINH039-19
U13	China, Jilin	1896	133	*T. mandshurica*	LMINH038-19
	China, Heilongjiang	1903	563	*Tilia* sp.	LMINH026-19
U14	China, Jilin	1896	278	*T. amurensis*	LMINH030-19
U15	China, Julin	1896	131	*T. amurensis*	LMINH032-19
U16	Russia, RFE (PK)	1936	563	*T. taquetii*	LMINH017-19
		1936	563	*T. taquetii*	LMINH042-19
U17	China, Julin	1896	277	*T. amurensis*	LMINH031-19
U18	China, Gansu	1911		*T. laetevirens*	LMINH036-19
U19	Russia, RFE (PK)	1941	563	*T. amurensis*	LMINH047-19
U20	Russia, ER (KO)	2014	563	*T. cordata*	LMINH002-19
U21	Russia, RFE (PK)	1987	563	*T. amurensis*	LMINH103-19
U22	Russia, RFE (PK)	1968	458	*T. mandshurica*	LMINH098-19
U23	China, Shandong	1924	563	*T. mandshurica*	LMINH022-19

*RFE – Russian Far East, PK – Primorsky Krai, KhK – Khabarovsk Krai, AO – Amur Oblast; ER – European Russia, KO – Kostroma Oblast. For China, provinces are indicated. The haplotypes in the lines colored in yellow correspond to the Cluster B, the remaining belong to the Cluster A (as shown in Figure 7).

Table S7. Number of examined herbarium specimens of *Tilia* spp. from the Northern Hemisphere stored in 20 herbarium depositaria in Eurasia*.

№	Herbarium institution (herbarium code), city, country	Number of herbarium specimens from	
		Palearctic	Nearctic
1	National Museum of Natural History (P), Paris, France	1426	2
2	Komarov Botanical Institute of the Russian Academy of Sciences (LECB), Saint Petersburg, Russia	2254	33
3	Royal Botanic Gardens (R), Kew, London, UK	1170	209
4	Naturalis Biodiversity Center (L), Leiden, the Netherlands	607	32
5	Natural History Museum (BM), London, UK	1128	86
6	Conservatory and Botanical Garden of the city of Geneva (G), Geneva, Switzerland	1025	5
7	Natural History Museum (W), Vienna, Austria	1057	59
8	Berlin Botanic Garden and Botanical Museum (B), Berlin, Germany	1034	26
9	Botanical Garden Zürich (ZT), Zurich, Switzerland	671	22
10	Moscow State University (MW), Moscow, Russia	761	22
11	Natural History Museum (FL), Florence, Italy	645	6
12	Royal Botanic Garden Edinburgh (E), Edinburgh, Scotland	1074	107
13	Principle Botanical Garden RAS (MHA), Moscow, Russia	539	23
14	Sapienza University of Rome, Rome (RO), Italy	321	0
15	National Museums Collection Center, Edinburgh, Scotland	7	0
16	Federal Scientific Center of the East Asia Terrestrial Biodiversity of the Far Eastern Branch of the Russian Academy of Sciences (VLA), Vladivostok, Russia	444	0
17	Botanic Garden–Institute of the Far Eastern Branch of the Russian Academy of Sciences (VBGI), Vladivostok, Russia	405	0
18	Tomsk State University (TK), Tomsk, Russia	253	6
19	Central Siberian Botanical Garden SB RAS of the Siberian Branch of the Russian Academy of Sciences (NS), Novosibirsk, Russia	180	0
20	Sukachev Institute of Forest SB RAS, Federal Research Center «Krasnoyarsk Science Center SB RAS» (KRF), Krasnoyarsk, Russia	8	0
	**In total:**	**15009**	**638**

*The depositaria are listed in an approximate order according to overall herbarium size.

Table S8. *Tilia* species and hybrids studied in the herbarium collections from different biogeographic regions*.

Lime species**	Number of lime species
**Palearctic**	
Tilia alba, ***T. amurensis,*** *T. apiculata, T. argentea, T. asplenifolia, T. aurea, T. blockiana, T. budensis, T. calvescens, T. caucasica, T. chinensis, T. chingiana, T. concinna,* ***T. cordata,*** *T. cordifolia, T. dasystyla, T. divaricata, T. dictyoneura, T. endochrysea, T. euchlora, T. flava, T. flavescens, T. floribunda, T. furedensis, T. gizellae, T. glabra, T. grandifolia, T. haringiana, T. haszlinszkyana, T. haynaldiana, T. henryana, T. heterophylla, T. insularis, T. intercedens, T. intermedia, T. intonsa, T. japonica, T. kiusiana, T. komarovi, T. koreana, T. laetevirens, T. latebracteata, T. latifolia, T. laxiflora, T. ledebourii, T. leptocarya,* ***T. mandshurica, T. maximowicziana****, T. microphylla, T. miqueliana, T. mollis, T. moltkei, T. mongolica, T. mutabilis, T. nasczokinii, T. neglecta, T. nickerlii, T. nobilis, T. oblique, T. oblongifolia T. obricularis, T. oliveri, T. oxycarpa, T. pallida, T. parvifolia, T. paucicostata, T. pekinensis, T. perneckensis, T. pilosa,* ***T. platyphyllos,*** *T. praecox, T. pubescens, T. pyramidalis, T. rubescens, T. rubra, T. rufa, T. ruprechtii, T. sibirica T. septemtrionalis, T. spectabilis, T. sphaerocarpa, T. stenocarpa, T. stohlii, T. subangulata, T. subflavescens, T. sublanata, T. sylvestris, T. sythensis,* ***T. taquetii,*** *T. tennifolia, T. tomentosa, T. trichoclados, T. truncate, T. tuan, T. tucekii, T. turbinata, T. ulmifolia, T. vestita, T. viridis, T. vitifolia, T. vulgaris*	101
Tilia spp. (hybrids)	> 80
**Nearctic**	
T. americana, *T. californiana, T. canadensis, T. caroliniana, T. floridana, T. glabra, T. heterophylla, T. lasioclada, T. leptophylla, T. littoralis, T. mexicana, T. michauxii, T. monticola, T. nuda, T. relicta, T. venulosa*	16
**In total (number of *Tilia* species examined, excluding hybrids):**	**117*****

*Based on examination of herbarium specimens in 20 herbarium depositaria (see Table S7). **The taxonomy of limes has been revised several times: *Tilia divaricata, T. koreana* and T. *taquetii* are presently considered as synonyms of *T. amurensis*; *T. sibirica* as a synonym of *T. cordata*; *T. pekinensis* as a synonym of *T. mandshurica*[42]. In our study, the names of lime species are provided as they were indicated on the labels of herbarium specimens. *Tilia* species marked in bold were dominant in herbaria (altogether they accounted 50% of all studied herbarium specimens).

Table S9. Diagnostic characters of leaf mines and pupae (based on pupal cremaster) in *Phyllonorycter* species feeding on limes, *Tilia* spp., in the Palearctic and Nearctic*.

Realm**	*Phyllonorycter* species	Leaf mine characteristics			Pupal cremaster
		Shape	Position	Folds on epidermis covering mine	
PALEARCTIC	*Ph. issikii*	Oval blotch	Lower side of the leaf usually between two secondary veins	Without folds	Roundish, with one pair of spines having wide base and curved outward tips
	Ph. messaniella	Oval blotch	Lower side of the leaf, usually between two veins	One distinct longitude fold which remains well visible in pressed leaves	Square-like, with two pairs of spines: one pair are long spines with relatively narrow base, another pair are short straight spine
NEARCTIC	*Ph. lucetiella*	Somewhat triangular blotch	Lower side of the leaf in the angle between the two veins	Without folds	Trapezoid, with two pairs of small spines: one pair curved outwards and another pair curved inward
	Ph. tiliacella	Irregular blotch	Usually on the upper side of the leaf, not associated with secondary veins	Without folds but with circular lines or dots of frass on the epidermis covering mine	Unknown

*According to published data[14,31,32,43]. **The listed species are originally known from these realms; *Ph. messaniella* is also known as an invasive species in Australasian realm, in particular in New Zealand and Australia.

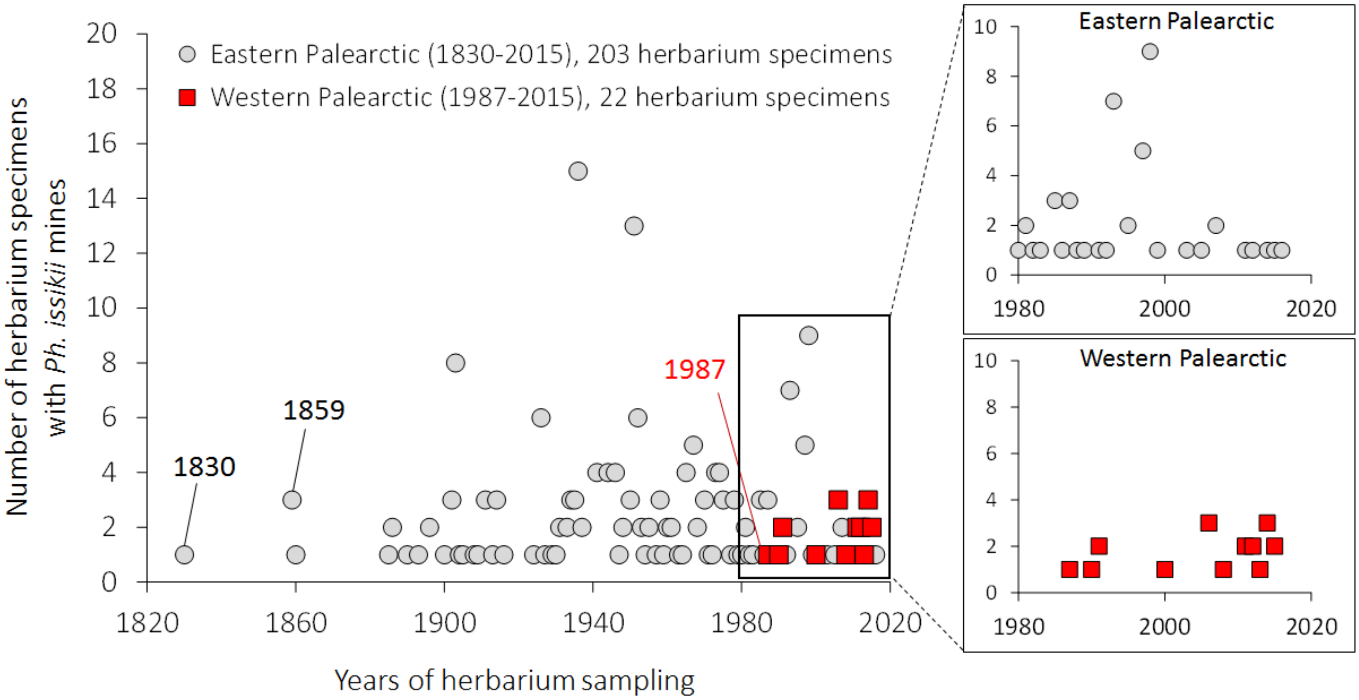

Figure S1. Dating of *Phyllonorycter issikii* mines in the lime herbarium specimens, *Tilia* spp., in the Palearctic: on the East, the putative native range (gray dots) and on the West, the putative invaded range (red dots). Zoomed in region shows the dispersal of overlapping findings on the East and on the West. The dots indicating the earliest mine records in herbaria in different parts of the Palearctic are supplied with the collection year. The earliest leaf mine was found in herbarium sample collected in Eastern Palearctic back in 1830 (191 years ago) (see also Figures 2a), whereas the earliest *Ph. issikii* mines were discovered in herbarium specimen collected in the Western Palearctic in 1987 (34 years ago). The oldest mine on the east (from the year 1830) was empty, whereas one of the mines found in 1859 (in the Russian Far East, Amur Oblast) carried larva which possession to *Ph. issikii* was confirmed by DNA barcoding (sample ID: LMINH119-19, see Figure 5). The earliest mine on the west (1987; Russia, The Urals, Chelyabinsk Oblast) also contained larva which was DNA barcoded and identified as *Ph. issikii* (sample ID: LMINH003-19, see Figure 5).

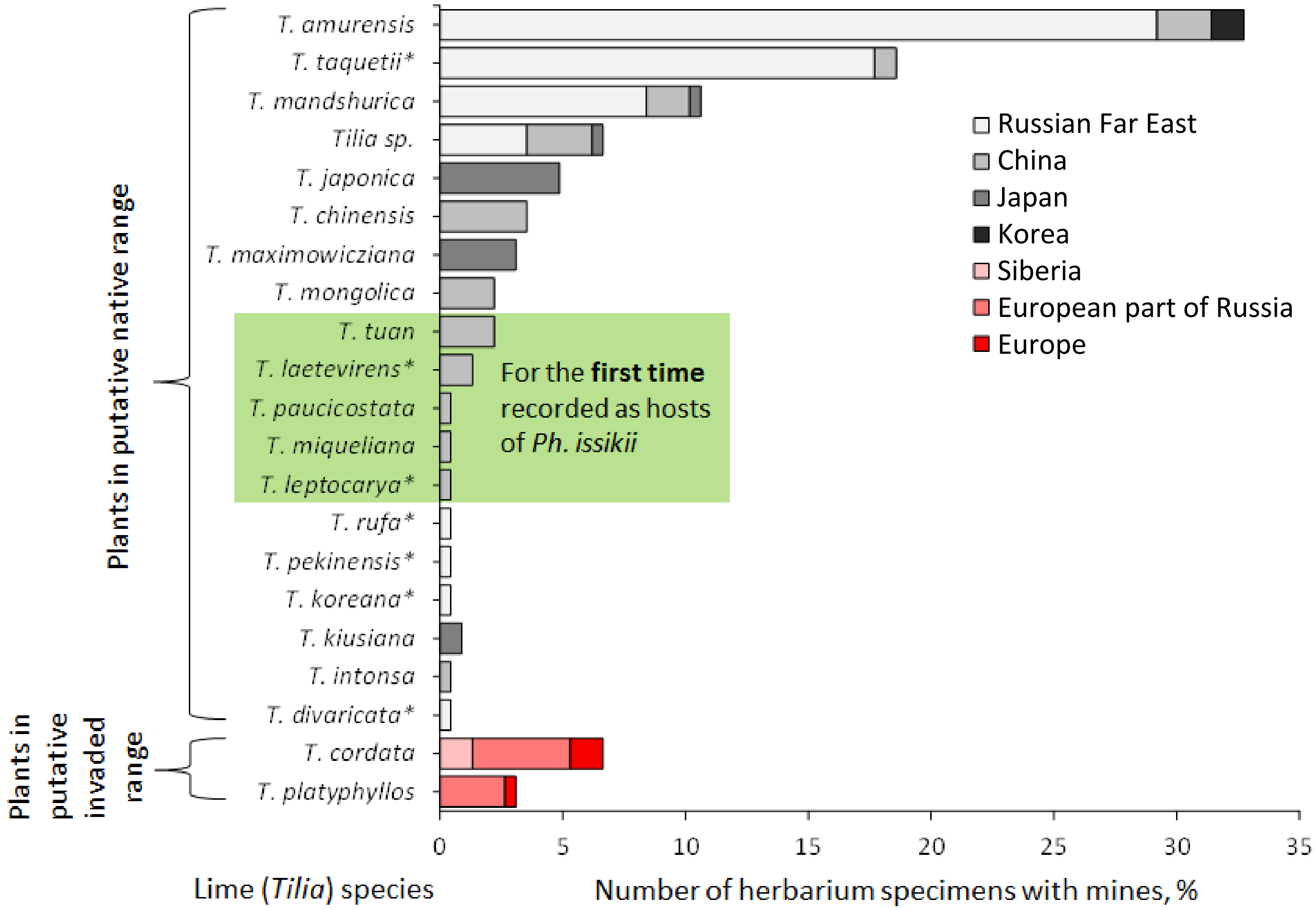

Figure S2. Host plants of *Phyllonorycter issikii* based on archival leaf mines found on 18 different *Tilia* species in historical herbaria collected in East Asia (putative native range) and two *Tilia* species in the Western Palearctic (invaded range). The species marked with an asterisk are presently known as junior synonyms: *Tilia taquetii, T. divaricata, T. koreana, T. rufa* are junior synonyms of *T. amurensis*; *T. laetevirens, T. leptocarya* and *T. pekinensis* are junior synonyms *of T. chinensis T. endochrysea* and *T. mandshurica* respectively.

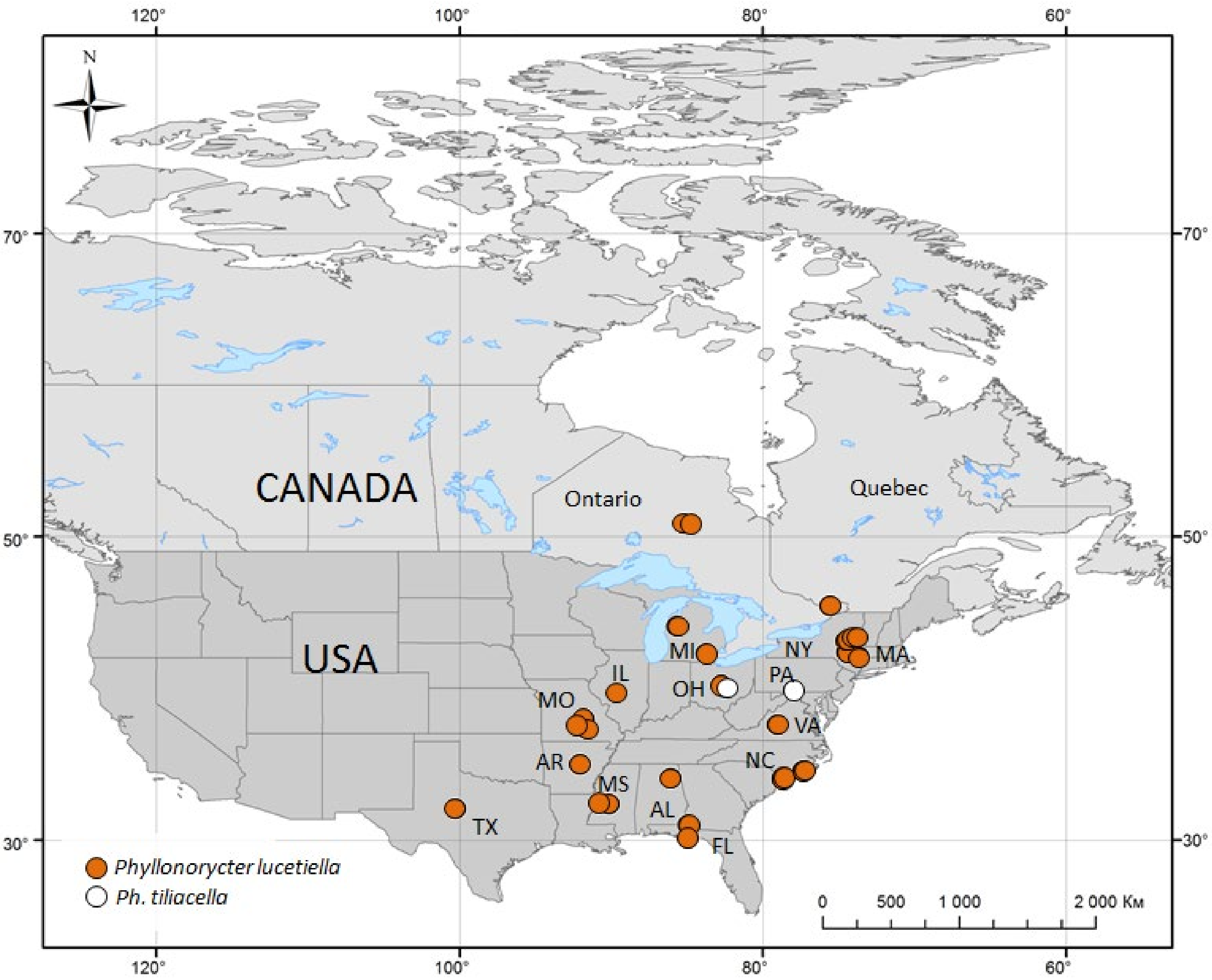

Figures S3. Geographical distribution of archival leaf mines of the North American micromoths, *Phyllonorycter lucetiella* and *Ph. tiliacella*, found in herbarium specimens of North American limes, *Tilia* spp., collected in the USA and Canada in the last 200 years. The states: AL – Alabama, AR – Arkansas, FL – Florida, IL – Illinois, MI – Michigan, MA – Massachusetts, MS – Mississippi, MO – Missouri, NC – North Carolina, NY – New York, OH – Ohio, PA – Pennsylvania, TX – Texas, VA – Virginia. Sampled *Tilia* species are listed in the Table S2. The map was generated using ArcGIS 9.3 (Release 9.3. New York St., Redlands, CA. Environmental Systems Research Institute, http://www.esri.com/software/arcgis/eval-help/arcgis-93).

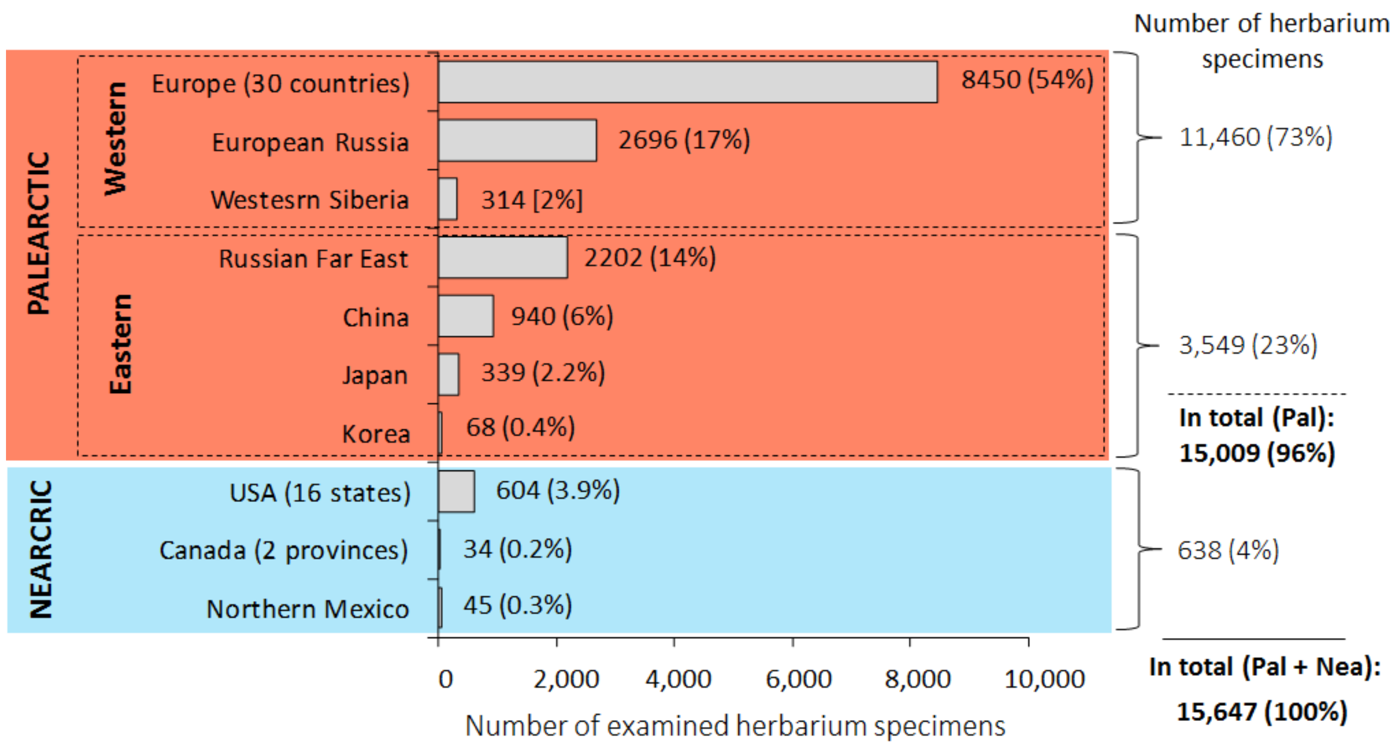

Figure S4. The number of herbarium specimens of limes, *Tilia* spp., sampled in the Northern Hemisphere in the last 253 years (1764–2016) involved in the study. The orange block indicates the Palearctic: Western, known as putative invaded range of *Phyllonorycter issikii* and Eastern, the putative native range. The blue block indicates the Nearctic, where *Ph. issikii* has not been documented so far. The number of herbarium specimens and their percentage (%) in a region or a country is given in parentheses next to the corresponding column and summarized for Western, Eastern Palearctics and Nearctic on the right side of the graph, where the total number of examined herbarium specimens in Palearctic (Pal) and in both Palearctic and Nearctic (Pal +Nea) is given.
